# Differences of blood cells, lymphocyte subsets and cytokines in COVID-19 patients with different clinical stages: a network meta-analysis

**DOI:** 10.1186/s12879-021-05847-9

**Published:** 2021-02-08

**Authors:** Wu Yan, Danrong Chen, Francis Manyori Bigambo, Hongcheng Wei, Xu Wang, Yankai Xia

**Affiliations:** 1 State Key Laboratory of Reproductive Medicine, Center for Global Health, School of Public Health, Nanjing Medical University, No.101 Longmian Road, Nanjing, Nanjing, 211166 China; 2 Key Laboratory of Modern Toxicology of Ministry of Education, School of Public Health, Nanjing Medical University, Nanjing, 211166 Jiangsu China; 3 Department of Endocrinology, Children’s Hospital of Nanjing Medical University, Nanjing, 210008 China

**Keywords:** COVID-19, Clinical stages, Blood cells, Lymphocyte subsets, Cytokines, Network meta-analysis

## Abstract

**Background:**

Due to the rapid spread of coronavirus disease 2019 (COVID-19) worldwide, it is necessary to ascertain essential immune inflammatory parameters that describe the severity of the disease and provide guidance for treatment. We performed network meta-analyses to determine differences in blood cells, lymphocyte subsets, and cytokines in COVID-19 patients with different clinical stages.

**Methods:**

Databases were systematically searched to May 2, 2020, and updated on June 1, 2020. Network meta-analyses were conducted via Stata 15.0, and the mean difference (MD) and its 95% CI were used as the effect values of the pooled analysis.

**Results:**

Seventy-one studies were included involving 8647 COVID-19 patients, White blood cell (WBC), neutrophil (NEUT), IL-6, and IL-10 counts increased significantly with worsening of the COVID-19, while lymphocyte (LYM) counts decreased. The levels of platelet (PLT), CD3^+^, CD4^+^, CD8^+^, and CD19^+^ cells in severe and critical patients were significantly lower than those in mild patients. IL-1β count was significantly elevated in critical patients.

**Conclusions:**

Immune suppression and inflammatory injury play crucial roles in the progression of COVID-19, and the identification of susceptible cells and cytokines provide guidance for the early and accurate treatment of COVID-19 patients.

**Supplementary Information:**

The online version contains supplementary material available at 10.1186/s12879-021-05847-9.

## Background

The coronavirus disease 2019 (COVID-19) is a pandemic disease caused by severe acute respiratory syndrome coronavirus 2 (SARS-CoV-2), a beta genus coronavirus that generally susceptible to humans. World Health Organization (WHO) COVID-19 Situation Report showed that up to June 8, 2020 more than 6,931,000 cases and 400,857 deaths were reported worldwide and there were 84, 634 total confirmed cases and 4645 deaths in China [1]. The patients with COVID-19 were classified into four groups based on the New Coronavirus Pneumonia Prevention and Control Program [2]: mild cases with mild clinical symptoms, and no pneumonia manifestation on imaging; moderate cases with any of fever, respiratory tract symptoms, and pneumonia manifestations in imaging; severe cases with respiratory rate ≥ 30 breath per minute, oxygen saturation ≤ 93% at rest state, arterial partial pressure of oxygen (PaO_2_)/ Oxygen concentration (FiO_2_) ≤ 300 mmHg and lung lesions progression > 50% manifestations in imaging within 24 to 48 h; and critical cases with any of respiratory failure requiring mechanical ventilation, shock, multiple organ failure requiring intensive care unit (ICU) treatment. These symptoms caused by COVID-19 infection were related to sustained responses of cytokines and chemokines (known as cytokine storm), which increased the incidence of immunity disorders and mortality [3].

Blood cells, lymphocytes, and cytokines levels can be used as important immune inflammatory parameters of adult patients with COVID-19 [4]. Lymphocytes and the subsets perform great roles in the maintenance of the function of the immune system. As with other infectious and immune diseases, viral infections can cause dysregulation of lymphocytes and their subsets [5]. T cells play a vital role in the clearance of the virus whereby CD3^+^ T cells and mature T cells activate CD4^+^ T cells and CD8^+^ T cells [6], cytotoxic T cells CD8^+^ produces molecules that eliminate the virus from the host [7], T helper cells (CD4^+^) help cytotoxic T cells and B cells (CD19^+^) to improve their capacity to clear pathogens [8]. Cytokines are small protein molecules such as IL-6, IL-10, and TNF-α secreted by T lymphocytes which play a great role in intercellular communications and immunomodulation function [9]. Although, continuous stimulation by the virus may cause T cells exhaustion a condition that may lead to a loss in cytokine production and decline in functions [10, 11] and multiple organ dysfunction [12].

Currently, most of the studies on immune inflammatory indicators in COVID-19 patients are single-centered and showed diverse results. Some studies have shown that compared with non-severe COVID-19, WBC, and NEUT in severe patients were significantly increased, while LYM was decreased [13–15]. In addition, the total counts of LYM subsets such as CD3^+^ T cells, CD4^+^ T cells, CD8^+^ T cells, and CD19^+^ B cells were substantially lower to both severe and non-severe COVID-19 patients [15, 16]. Moreover, cytokines such as IL-6, IL-10, and TNF-α were revealed to increase with the severity of the diseases in COVID-19 patients [15, 17–19]. A study by Zhu et al. confirmed the predictive value of immune-inflammation parameters that IL-6 played a pivotal role in the severity of COVID-19 and had a potential value for monitoring the process of severe cases [20]. However, some studies reported controversial results that the level of WBC in severe patients was lower than the mild [21–23], but the LYM tended to increase with the deterioration of the disease [4, 24, 25]. Furthermore, studies focusing on immune inflammatory parameters of COVID-19 patients with different clinical stages rarely consider whether the severity of the disease varies in each age group. In addition, there is still a controversy on how their inflammation parameters change.

In the presence of this rapidly emerging novel infectious disease, it is necessary to identify immune inflammatory parameters that could predict the disease severity and the prognosis essentially for guiding clinical care. Therefore, we categorized COVID-19 patients into mild, severe, and critical groups as well as performed network meta-analysis to compare the difference in the blood cells, lymphocyte subsets, and cytokines at different clinical stages.

## Methods

### Search strategy

The network meta-analysis was performed based on the Preferred Reporting Items for Systematic Review and Meta-Analysis (PRISMA) guidelines (Additional file 1: Table S1). We systematically retrieved relevant articles from PubMed, Embase, Web of Science, Cochrane Library, China national knowledge internet (CNKI), Wanfang Database, and China biology medicine databases (CBM) for papers published before May 2, 2020, and updated on June 1, 2020. The references of included studies were also retrieved to avoid omissions. The search strategy was shown in (Additional file 1: Table S2).

### Selection criteria

The inclusion criteria were: (1) COVID-19 patients who tested positive for nucleic acid. (2) COVID-19 patients whose clinical stages were sorted based on the currently accepted clinical stages. The exclusion criteria were: (1) Animal trials, review, conference abstracts, case reports, and editorial materials. (2) Specific populations such as children and pregnant women. (3) Incomplete full text or non-conforming data. (4) Different studies with reduplicated populations.

### Study selection and data extraction

Two reviewers independently retrieved the articles and directly imported them into Endnote X9 to manage search records. We conducted screening of the titles and abstracts of the search records based on the inclusion and exclusion criteria. Full texts of the eligible articles were also retrieved for further confirmation. Then, relevant data were extracted from selected studies, included the first author name, research time, region, sample size, gender, mean age, gender, clinical stages and case number, the type of immune inflammatory parameters, and quality scores.

### Quality assessment

The methodological qualities of the included cross-sectional studies were assessed based on the 11-items checklist recommended by the agency for healthcare research and quality (AHRQ). If the answer was ‘yes’, it was given one score; if the answer was ‘no’ or ‘unclear’, this item scored ‘0’. The total number of the scores presented the quality of the included studies were classified into three levels: 8 to 11 was regarded as high quality; 4 to 7 as moderate quality; and 0 to 3 as low quality [26].

### Statistical analysis

In our study, Stata15.0 was used for all data analyses. For part of the data presented in the median (interquartile range) formats, we estimated them to the mean ± standard deviation (SD) using an online data converter compiled by Wan and Luo et al. *(**http://www.math.hkbu.edu.hk/~tongt/papers/median2mean.html*). The converter considered the sample size and proved to be the closest to the actual estimation method [27, 28]. Since the measurement units of the indicators were consistent in each study, the mean difference (MD) and its 95% CI were used as the results of the pooled analysis. The “*network*” package was used for conducting the meta-analysis, performing consistency and inconsistency tests, and using the “network forest” function to draw forest maps. A node-splitting method was applied to assess the inconsistency between direct and indirect comparisons, which would affect the authenticity of the network meta-analysis, so it was necessary to detect the inconsistencies. For the closed loop formed by the three measures, the inconsistency between direct and indirect evidence could be directly compared. The closed loop formed by the four measures should be divided into two closed triangular loops to analyze the inconsistency between direct evidence and indirect evidence [29]. After fitting the consistency model, the mean values of the pooled effects in each group were ranked from the largest to the smallest, and the SUCRA values were calculated and compared in pairs. Finally, we performed a subgroup analysis based on the mean ages.

## Results

### Literature selection

**Fig. 1:**
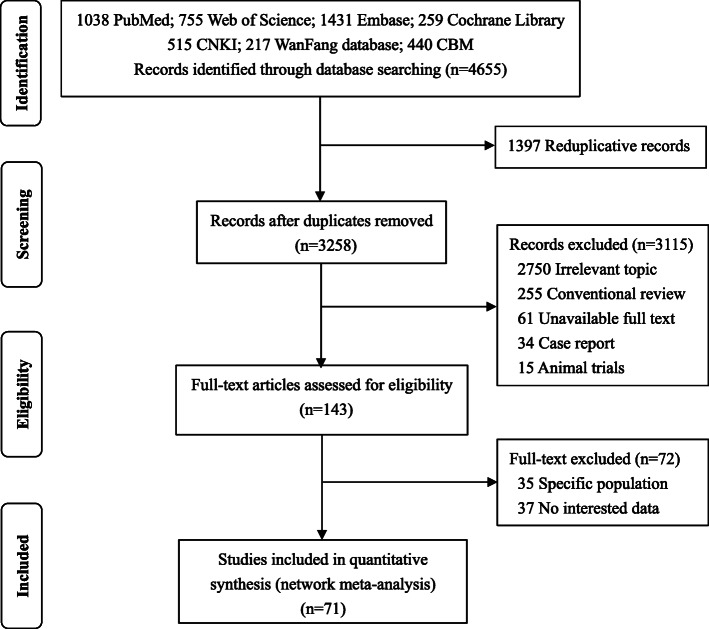
The process and procedures of study screening

A total of 4, 655 candidate papers were initially identified, including 1172 Chinese and 3483 English articles. After reviewing the titles and abstracts, 143 studies were considered for further review. Of these studies, 35 were focused on a specific population, 37 had no interesting data, and eventually, 71 studies met inclusion criteria for our meta-analysis. The detailed selection process was shown in Fig. 1.

### Characteristics of the included studies

A total of 8, 647 COVID-19 patients from 14 regions in China and Daegu, Korea were included in our study. The sample size ranged from 10 to 548, and the average age of the confirmed patients ranged from 35 years to 66.9 years. Forty-four of the 71 studies focused on both mild and severe patients, while the remaining studies included patients in mild, severe, and critical groups. In the network meta-analysis, we classified COVID-19 patients into mild, severe, and critical groups based on the severity of the disease. The characteristics of the included studies were shown in (Additional file 1: Table S3).

### Quality of included studies

The quality assessment checklist ensures that the included studies meet the quality requirements. The average quality score of the included studies was 7.86 ± 0.86, with 47 high quality studies and 24 moderate quality studies based on grades. The results of the AHRQ quality were shown in (Additional file 1: Table S3).

### Network meta-analysis

#### Blood cells

WBC counts were reported in 58 studies involving 7210 COVID-infected patients, of which 18 studies compared WBC levels in mild, severe, and critical groups, and 40 studies reported mild and severe cases. In the network meta-analysis, we found that the WBC counts in the severe group (MD = 1.10, 95% CI: 0.68 to 1.53) and the critical group (MD = 2.26, 95% CI: 1.57 to 2.95) were significantly higher than those in the mild group. Further comparison showed that the critical group was higher than the severe group, and the difference was also statistically significant.

LYM levels of different clinical stages were reported in 59 studies, and the lymphocyte levels in the severe and critical patients were significantly lower (MD = − 0.37, 95% CI: − 0.44 to − 0.31; MD = − 0.58, 95% CI: − 0.68 to − 0.48, respectively) than the mild, and the reduction was more significant in the critical group. Compared to COVID-19 mild patients, NEUT levels in both severe patients (MD = 1.20, 95% CI: 0.65 to 1.75) and critical patients (MD = 2.76, 95% CI: 1.92 to 3.60) were increased significantly, and the NEUT count in critical patients (MD = 1.56, 95% CI: 0.70 to 2.42) was higher than that in severe patients. PLT were significantly higher in severe and critical patients than in the mild (MD = − 18.87, 95% CI: − 27.86 to − 9.88; MD = − 30.13, 95% CI: − 45.24 to − 15.03, respectively). The concentration of HB in the severe patients (MD = − 3.81, 95% CI: − 5.43 to − 2.19) was significantly lower than that of the mild group, but no statistically significant difference was found in other groups. Only one of the 51 included studies reported basophil lymphocytes, so we did not perform network meta-analyses on them (Table 1).

**Table 1:** Network meta-analysis of blood cell levels in COVID-19 patients with different clinical stages MD (95%CI)

Comparisons	WBC (× 10^9^/L)	LYM (×10^9^/L)	NEUT (×10^9^/L)	MONO (×10^9^/L)	PLT (×10^9^/L)	HB (g/L)
Severe VS. Mild ^a^	**1.10 (0.68, 1.53)**	**−0.37 (− 0.44, − 0.31)**	**1.20 (0.65, 1.75)**	−0.03 (− 0.08, 0.01)	**−18.87 (− 27.86, − 9.88)**	**−3.81 (− 5.43, − 2.19)**
Critical VS. Mild ^a^	**2.26 (1.57, 2.95)**	**−0.58 (− 0.68, − 0.48)**	**2.76 (1.92, 3.60)**	−0.04 (− 0.10, 0.02)	**−30.13 (− 45.24, − 15.03)**	−3.99 (− 8.75, 0.76)
Critical VS. Severe ^a^	**1.16 (0.46, 1.86)**	**−0.21 (− 0.31, − 0.10)**	**1.56 (0.70, 2.42)**	−0.01 (− 0.08, 0.06)	−11.26 (− 26.74, 4.21)	−0.18 (− 5.04, 4.67)
Inconsistency *P*-value	0.070	0.150	0.115	0.871	0.793	0.484

^a^When comparing, the groups in the later positions were taken as the reference

Abbreviations: *WBC* white blood cell, *LYM* lymphocyte, *NEUT* neutrophil, *PLT* platelet, *HB* hemoglobin

#### Lymphocyte subsets

Some of the included studies explored the levels of LYM subsets in patients with different clinical stages of COVID-19, of which the largest number was CD4^+^ T cells. A total of 2216 patients from 14 studies reported the differences in CD4^+^ T cells counts, 12 studies involved 2091 patients reported about CD8^+^ T cells levels. The results of network meta-analysis showed that the count of CD3^+^ T cells were significantly lower in the severe group and the critical group (MD = − 389.02, 95% CI: − 549.59 to − 228.45; MD = − 479.87, 95% CI: − 696.64 to − 263.10, respectively). The trend of the critically ill group was lower than that of the severely ill group, but there was no statistically significant difference (MD = − 90.85, 95% CI: − 305.37 to 123.67). The counts of LYM subsets such as CD4^+^ T cells, CD8^+^ T cells, and CD19^+^ B cells also showed consistent results. Additionally, the count of CD16^+^ CD56^+^ cells was significantly decreased in the critical group than that of the mild group (MD = − 57.68, 95% CI: − 101.37 to 14.00), but there was no statistically significant difference in the severe group compared to the mild group and the critical group (Table 2).

**Table 2:** Network meta-analysis of lymphocyte subsets levels in COVID-19 patients with different clinical stages MD (95%CI)

Comparisons	CD3^+^ (/μL)	CD4^+^ (/μL)	CD8^+^ (/μL)	CD19^+^ (/μL)	CD16^+^ CD56^+^ (/μL)
Severe VS. Mild ^a^	**−389.02 (− 549.59, − 228.45)**	**−198.74 (− 262.12, −135.35)**	**−127.49 (− 173.75, − 81.24)**	**− 134.32 (− 203.92, − 64.71)**	− 23.27 (− 55.79, 9.25)
Critical VS. Mild ^a^	**−479.87 (− 696.64, − 263.10)**	**− 262.18 (− 356.76, − 167.60)**	**− 159.06 (− 223.91, − 94.22)**	**−173.38 (− 289.84, − 56.91)**	**−57.68 (− 101.37, − 14.00)**
Critical VS. Severe ^a^	−90.85 (− 305.37, 123.67)	−63.44 (− 157.51, 30.62)	− 31.57 (− 96.09, 32.95)	−39.06 (− 155.12, 76.99)	− 34.41 (− 78.91, 10.08)
Inconsistency *P*-value	0.135	0.081	0.049	0.279	0.639

^a^When comparing, the groups in the later positions were taken as the reference

Abbreviation: *CD* cluster of differentiation

#### Cytokines

Three studies compared IL-1β levels of COVID-19 patients were included in the network meta-analysis. The results showed that IL-1β count in the critical group (MD = 0.15, 95% CI: 0.01 to 0.30) was significantly higher than those in the mild group, while the comparison between severe and mild groups was not statistically significant. It is worth noting that IL-6 was the most reported cytokine among COVID-19 patients included in the study, and a total of seventeen IL-6 relevant studies were included. There were significant differences in IL-6 levels among patients with different clinical stages. Compared with the mild group, 25.70 ng/L (95% CI: 9.99 to 41.41) was increased in the severe group and 56.90 ng/L (95% CI: 36.29 to 77.52) in the critical group, respectively. At the same time, the critical group was also significantly higher than the severe group. Furthermore, IL-10 and IL-6 counts were similar in patients with different clinical stages. The level of TNF-α were not statistically significant differences between the three groups. Some additional cytokines such as IL-2, IL-8, etc., were only mentioned in a few studies, so it was difficult to perform network meta-analysis (Table 3).

**Table 3:** Network meta-analysis of cytokines levels in COVID-19 patients with different clinical stages MD (95%CI)

Comparisons	IL-1β (ng/L)	IL-6 (ng/L)	IL-10 (ng/L)	TNF-α (ng/L)
Severe VS. Mild ^a^	0.07 (−0.05, 0.20)	**25.70 (9.99, 41.41)**	**1.42 (0.31, 2.54)**	0.28 (−2.41, 2.97)
Critical VS. Mild ^a^	**0.15 (0.01, 0.30)**	**56.90 (36.29, 77.52)**	**3.93 (2.23, 5.63)**	−0.11 (−3.49, 3.26)
Critical VS. Severe ^a^	0.08 (−0.10, 0.26)	**31.21 (10.59, 51.82)**	**2.51 (0.54, 4.47)**	−0.40 (−3.79, 3.00)
Inconsistency *P*-value	0.545	0.408	0.510	0.993

^a^When comparing, the groups in the later positions were taken as the reference

Abbreviations: *IL* interleukin, *TNF* tumor necrosis factor

Forest maps and funnel plots of all the results were presented (Additional file 1: FigureS1-S30). Subgroup analysis showed that the group below the average age (< 52 years) was mostly mild and severe, with a small proportion of severe cases. In the above-average age group (≥52 years), most of the variation in immune inflammatory parameters were closer to the overall analysis (Additional file 1: Table S4). The evaluation of the inconsistency between direct and indirect comparisons by node-splitting model was not statistically significant to most of the studies (*P* > 0.05) (Additional file 1: Table S5).

## Discussion

In this study, we systematically reviewed the available evidence and conducted a network meta-analysis to describe the differences in the blood cell, lymphocyte subsets, and cytokine levels in patients suffered from COVID-19 at different clinical stages. Our results showed that with the severity of COVID-19 worsens, the count of WBC, NEUT, IL-6, and IL-10 increased significantly, while LYM levels decreased. Compared with the mild group, the levels of PLT, CD3^+^ T cells, CD4^+^ T cells, CD8^+^ T cells, and CD19^+^ B cells were significantly decreased in the severe and critical patients. IL-1β was significantly increased in critically ill patients.

Our results were consistent with most studies focusing on immune inflammatory parameters abnormalities in COVID-19 patients, documented the higher WBC and NEUT count in the severe group than in the non-severe group [18, 30–32]. With the aggravation of the disease, COVID-19 patients are susceptible to bacterial and fungal infections due to low autoimmunity, and the increase in WBC and NEUT counts reflecting their inflammation levels are higher than those of the mild patients [33]. The increase in NEUT counts may be associated with cytokine storms caused by viral invasion [30]. LYM decrease was associated with the increase in the disease severity in COVID-19 patients. The possible mechanism of LYM reduction was that the virus attacks target cells, directly damage cells, and the viral infection caused non-specific damage, immune cells enter the activated state and participate in the antiviral process, leading to severe injury and apoptosis. Therefore, it can be considered that the supplement of LYM may be the key to the recovery of COVID-19 patients [34].

In our study, we found that LYM was reduced in COVID-19 patients, especially in the critically ill group. Typical T lymphocytes (CD3^+^) were mainly reduced, while no significant variation was observed in NK cells (CD16^+^ CD56^+^), which have been confirmed in other studies [33, 35]. LYM subsets are essential for maintaining immune system function and eliminating viruses and are often altered after viral infection. The SARS-CoV, MERS-CoV, and SARS-CoV-2 of the coronavirus family may all cause a decrease in the count of LYM and its subset of infected patients [19], which may be due to the homology, similar genes, viral structure, and pathogenesis among the three [36]. At present, limited autopsy and pathological findings have found that the spleen, lymph nodes, and other lymphoid tissues of COVID-19 infected patients were necrotic, and lymphocyte infiltration was observed in alveolar septum [33], presumably be caused by direct virus attack or immune injury mediated by it. Isolation of lymphocytes in the lung may be another potential cause, but the specific mechanism needs to be further explored.

Meanwhile, as the severity of COVID-19 worsens, the concentrations of inflammatory cytokines are getting higher. A recently published meta-analysis showed that IL-6 levels in patients with severe COVID-19 were higher than those in non-severe COVID-19 patients (MD: 38.6 ng/L, 95% CI: 24.3–52.9 ng/L) [37], the trend of difference was consistent with our study. In many cases, elevated IL-6 has been demonstrated to have different degrees of inflammation [38]. The pathophysiological characteristics of COVID-19 are severe inflammation and chemokine storms, which may be the driving factors behind acute lung injury and acute respiratory distress syndrome [39], as well as the main consequence of IL-6 elevation [30, 40]. Mild patients had slightly higher cytokine levels or were in the normal range, which means there was no excessive inflammatory response in the body. However, serum levels of various cytokines in critical patients were significantly elevated, suggesting that the body is in an over-immune state during the progression of the disease, a large number of inflammatory cells and inflammatory mediators have been released in critical patients [39, 41].

Our quantitative analysis results were consistent with most of the published research papers, but we were also aware of some inconsistencies. We found that the number of COVID-19 patients included in some studies was small, and the sample size distributions of the three groups were greatly varied. In addition, the age of the patients in the severe group was generally higher than that in the mild group, which is in line with the real world. For example, there were 12 cases in the study of Liu et al., among which only 3 patients had critical symptoms [42]. Moreover, a study by Xiao et al. [21], accounted for higher weight in our network meta-analysis due to the large sample size. However, the average age of the study subjects was relatively young, and the proportion of the critical group was less than 9% of the total patients, which may explain the inconsistency.

To the best of our knowledge, this is the first network meta-analysis that systematically reviewed the differences in blood cells, lymphocyte subsets, and cytokine levels among COVID-19 patients with different clinical stages. First, we completed the comparisons of immune inflammatory parameters between any two groups among the mild, severe, and critical patients. Most of the included studies were divided into mild and severe groups, but our analysis of the critical cases was not limited, which was difficult to accomplish in the conventional meta-analysis. In addition, we analyzed as many immune-related cells and cytokines as possible to demonstrate potential relationships and differences. Importantly, we found that patients who were above-average age had a higher proportion of critical events and stronger changes in their immune inflammatory parameters. It is worth mentioning that the total number of patients included in the network meta-analysis was large, and the effect value of the pooled analysis was stable, so the results can be considered reliable and powerful. Some limitations of our studies should be noted. Firstly, the included studies were cross-sectional study and the cases were hospitalized cases, no further conditions of the patients were reported. Secondly, the mean age of the patients in each study varied widely, and subjects of different age groups have varied physical conditions, which may also affect the changes of immune inflammatory parameters during illness. Besides, the sample size of some studies was small, which may reduce the power of the studies. Ultimately, most of the studies included were performed in China, and measurements may be varied across laboratories.

## Conclusions

The immune suppression and inflammatory injury play crucial roles in the progression of COVID-19, and the identification of susceptible cells and cytokines can also guide the development of accurate treatment plans for patients with different clinical stages. The results reminded us to emphasize the cytokine storm in the progression of COVID-19. In the future, large-scale, long-term research should be devoted to confirm the results of our network meta-analysis, identify other immune inflammatory parameters of COVID-19 serious infection or poor prognosis, and clarify the underlying mechanisms of their relationship.

## Supplementary Information

**Additional file 1: Table S1** PRISMA checklist. **Table S2** Search strategies. **Table S3** Characteristics of the articles included in our meta-analysis. **Fig. S1** Forest maps of white blood cell (WBC) comparison in COVID-19 patients with different clinical stages. * represents statistically significant differences (*P* < 0.05). **Fig. S2** Forest maps of lymphocyte (LYM) comparison in COVID-19 patients with different clinical stages. * represents statistically significant differences (*P* < 0.05). **Fig. S3** Forest maps of neutrophil (NEUT) comparison in COVID-19 patients with different clinical stages. * represents statistically significant differences (*P* < 0.05). **Fig. S4** Forest maps of monocytes (MONO) comparison in COVID-19 patients with different clinical stages. * represents statistically significant differences (*P* < 0.05). **Fig. S5** Forest maps of platelet (PLT) comparison in COVID-19 patients with different clinical stages. * represents statistically significant differences (*P* < 0.05). **Fig. S6** Forest maps of hemoglobin (HB) comparison in COVID-19 patients with different clinical stages. * represents statistically significant differences (*P* < 0.05). **Fig. S7** Forest maps of cluster of differentiation 3 (CD3^+^) comparison in COVID-19 patients with different clinical stages. * represents statistically significant differences (*P* < 0.05). **Fig. S8** Forest maps of cluster of differentiation 4 (CD4^+^) comparison in COVID-19 patients with different clinical stages: *represents statistically significant differences (*P* < 0.05). **Fig. S9** Forest maps of cluster of differentiation 8 (CD8^+^) comparison in COVID-19 patients with different clinical stages: *represents statistically significant differences (*P* < 0.05). **Fig. S10** Forest maps of cluster of differentiation 19 (CD19^+^) comparison in COVID-19 patients with different clinical stages: *represents statistically significant differences (*P* < 0.05). **Fig. S11** Forest maps of cluster of differentiation 16^+^ 56^+^ (CD16^+^ CD56^+^) comparison in COVID-19 patients with different clinical stages: *represents statistically significant differences (*P* < 0.05). **Fig. S12** Forest maps of interlcukin-1β (IL-1β) comparison in COVID-19 patients with different clinical stages. * represents statistically significant differences (*P* < 0.05). **Fig. S13** Forest maps of interlcukin-6 (IL-6) comparison in COVID-19 patients with different clinical stages. * represents statistically significant differences (*P* < 0.05). **Fig. S14** Forest maps of interlcukin-10 (IL-10) comparison in COVID-19 patients with different clinical stages. * represents statistically significant differences (*P* < 0.05). **Fig. S15** Forest maps of tumor necrosis factor-α (TNF-α) comparison in COVID-19 patients with different clinical stages. * represents statistically significant differences (*P* < 0.05). **Fig. S16** Funnel plot, WBC. **Fig. S17** Funnel plot, LYM. **Fig. S18** Funnel plot, NEUT. **Fig. S19** Funnel plot, MONO. **Fig. S20** Funnel plot, PLT. **Fig. S21** Funnel plot, HB. **Fig. S22** Funnel plot, CD3^+^. **Fig. S23** Funnel plot, CD4^+^. **Fig. S24** Funnel plot, CD8^+^. **Fig. S25** Funnel plot, CD19^+^. **Fig. S26** Funnel plot, CD16^+^ CD56^+^. **Fig. S27** Funnel plot, IL-1β. **Fig. S28** Funnel plot, IL-6. **Fig. S29** Funnel plot, IL-10. **Fig. S30** Funnel plot, TNF-α. **Table S4** Subgroup analysis of immune-inflammatory parameters in COVID-19 patients with different clinical types. **Table S5** Comparison of direct, indirect, and network meta-analyses results.

## Data Availability

All data related to the present study are available in the manuscript and supplementary files.

## Abbreviations

COVID-19: Coronavirus disease 2019
WBC: White blood cell
LYM: Lymphocyte
NEUT: Neutrophil
PLT: Platelet
HB: Hemoglobin
CD: Cluster of differentiation
IL: Interleukin
TNF: Tumor necrosis factor
IQR: Interquartile range
MD: Mean difference
SD: Standard deviation
AHRQ: Agency for healthcare research and quality
