# Supplementary material for: Differences of blood cells, lymphocyte subsets and cytokines in COVID-19 patients with different clinical stages: a network meta-analysis
Source: BMC Infect Dis. 2021 Feb 8;21:156. doi: 10.1186/s12879-021-05847-9 (PMC7868864; doi:10.1186/s12879-021-05847-9)
Supplement: Supplementary file 1 — Additional file 1: Table S1 PRISMA checklist. Table S2 Search strategies. Table S3 Characteristics of the articles included in our meta-analysis. Fig. S1 Forest maps of white blood cell (WBC) comparison in COVID-19 patients with different clinical stages. * represents statistically significant differences (P < 0.05). Fig. S2 Forest maps of lymphocyte (LYM) comparison in COVID-19 patients with different clinical stages. * represents statistically significant differences (P < 0.05). Fig. S3 Forest maps of neutrophil (NEUT) comparison in COVID-19 patients with different clinical stages. * represents statistically significant differences (P < 0.05). Fig. S4 Forest maps of monocytes (MONO) comparison in COVID-19 patients with different clinical stages. * represents statistically significant differences (P < 0.05). Fig. S5 Forest maps of platelet (PLT) comparison in COVID-19 patients with different clinical stages. * represents statistically significant differences (P < 0.05). Fig. S6 Forest maps of hemoglobin (HB) comparison in COVID-19 patients with different clinical stages. * represents statistically significant differences (P < 0.05). Fig. S7 Forest maps of cluster of differentiation 3 (CD3+) comparison in COVID-19 patients with different clinical stages. * represents statistically significant differences (P < 0.05). Fig. S8 Forest maps of cluster of differentiation 4 (CD4+) comparison in COVID-19 patients with different clinical stages: *represents statistically significant differences (P < 0.05). Fig. S9 Forest maps of cluster of differentiation 8 (CD8+) comparison in COVID-19 patients with different clinical stages: *represents statistically significant differences (P < 0.05). Fig. S10 Forest maps of cluster of differentiation 19 (CD19+) comparison in COVID-19 patients with different clinical stages: *represents statistically significant differences (P < 0.05). Fig. S11 Forest maps of cluster of differentiation 16+ 56+ (CD16+ CD56+) comparison in COVID-19 patients [file 12879_2021_5847_MOESM1_ESM.docx]

**Differences of** **Blood Cells, Lymphocyte Subsets and Cytokines in COVID-19 Patients with Different Clinical Stages: A Network Meta-analysis**

Wu Yan ^1, 2*^, Danrong Chen ^1, 2*^, Francis Manyori Bigambo ^1,^ ^2*^, Hongcheng Wei ^1, 2^, Xu Wang ^1, 2#^, Yankai Xia ^1, 2#^

^1^ State Key Laboratory of Reproductive Medicine, Center for Global Health, School of Public Health, Nanjing Medical University, Nanjing 211166, China

^2^ Key Laboratory of Modern Toxicology of Ministry of Education, School of Public Health, Nanjing Medical University, Nanjing, Jiangsu 211166, China

**APPENDIX**

1. PRISMA checklist **(Table S1)**
2. Search strategies **(Table S2)**
3. Characteristics of the articles included in our meta-analysis **(Table S3)**
4. Forest maps of blood cells, lymphocyte subsets and cytokines comparison in COVID-19 patients with different clinical stages **(Figure S1-S15)**
5. Funnel plots **(Figure S16-S30)**
6. Subgroup analysis of immune-inflammatory parameters in COVID-19 patients with different clinical types (**Table S4**)
7. Comparison of direct, indirect, and network meta-analyses results (**Table S5**)

**Table S1** PRISMA checklist

| **Section/topic** | **#** | | **Checklist item** | | | **Reported on page #** |
| --- | --- | --- | --- | --- | --- | --- |
| **TITLE** | | | | | |  |
| Title | 1 | | Identify the report as a systematic review, meta-analysis, or both. | | | Title, |
| **ABSTRACT** | | | | | |  |
| Structured summary | 2 | | Provide a structured summary including, as applicable: background; objectives; data sources; study eligibility criteria, participants, and interventions; study appraisal and synthesis methods; results; limitations; conclusions and implications of key findings; systematic review registration number. | | | Abstract, |
| **INTRODUCTION** | | | | | |  |
| Rationale | 3 | | Describe the rationale for the review in the context of what is already known. | | | Background, paragraph 1-3 |
| Objectives | 4 | | Provide an explicit statement of questions being addressed with reference to participants, interventions, comparisons, outcomes, and study design (PICOS). | | | Background, paragraph 5 |
| **METHODS** | | | | | |  |
| Protocol and registration | 5 | | Indicate if a review protocol exists, if and where it can be accessed (e.g., Web address), and, if available, provide registration information including registration number. | | | N/A |
| Eligibility criteria | 6 | | Specify study characteristics (e.g., PICOS, length of follow-up) and report characteristics (e.g., years considered, language, publication status) used as criteria for eligibility, giving rationale. | | | Methods, paragraph 5 |
| Information sources | 7 | | Describe all information sources (e.g., databases with dates of coverage, contact with study authors to identify additional studies) in the search and date last searched. | | | Methods, paragraph 1 |
| Search | 8 | | Present full electronic search strategy for at least one database, including any limits used, such that it could be repeated. | | | Additional files  Search strategies |
| Study selection | 9 | | State the process for selecting studies (i.e., screening, eligibility, included in systematic review, and, if applicable, included in the meta-analysis). | | | Methods, paragraph 2 |
| Data collection process | 10 | | Describe method of data extraction from reports (e.g., piloted forms, independently, in duplicate) and any processes for obtaining and confirming data from investigators. | | | Methods, paragraph 3 |
| Data items | 11 | | List and define all variables for which data were sought (e.g., PICOS, funding sources) and any assumptions and simplifications made. | | | Methods, paragraph 3 |
| Risk of bias in individual studies | 12 | | Describe methods used for assessing risk of bias of individual studies (including specification of whether this was done at the study or outcome level), and how this information is to be used in any data synthesis. | | | Methods, paragraph 4 |
| Summary measures | 13 | | State the principal summary measures (e.g., risk ratio, difference in means). | | | Methods, paragraph 5 |
| Synthesis of results | 14 | | Describe the methods of handling data and combining results of studies, if done, including measures of consistency (e.g., I^2^) for each meta-analysis. | | | Methods, paragraph 5 |
| Risk of bias across studies | | 15 | | Specify any assessment of risk of bias that may affect the cumulative evidence (e.g., publication bias, selective reporting within studies). | Methods, paragraph 5 | |
| Additional analyses | | 16 | | Describe methods of additional analyses (e.g., sensitivity or subgroup analyses, meta-regression), if done, indicating which were pre-specified. | Methods, paragraph 5 | |
| **RESULTS** | | | | |  | |
| Study selection | | 17 | | Give numbers of studies screened, assessed for eligibility, and included in the review, with reasons for exclusions at each stage, ideally with a flow diagram. | Results, paragraph 1 | |
| Study characteristics | | 18 | | For each study, present characteristics for which data were extracted (e.g., study size, PICOS, follow-up period) and provide the citations. | Results, paragraph 2 | |
| Risk of bias within studies | | 19 | | Present data on risk of bias of each study and, if available, any outcome level assessment (see item 12). | Results, paragraph 3 | |
| Results of individual studies | | 20 | | For all outcomes considered (benefits or harms), present, for each study: (a) simple summary data for each intervention group (b) effect estimates and confidence intervals, ideally with a forest plot. | Results, paragraph 4-7 | |
| Synthesis of results | | 21 | | Present results of each meta-analysis done, including confidence intervals and measures of consistency. | Results, paragraph 4-7 | |
| Risk of bias across studies | | 22 | | Present results of any assessment of risk of bias across studies (see Item 15). | Additional Files | |
| Additional analysis | | 23 | | Give results of additional analyses, if done (e.g., sensitivity or subgroup analyses, meta-regression [see Item 16]). | Additional Files  Table S4 | |
| **DISCUSSION** | | | | |  | |
| Summary of evidence | | 24 | | Summarize the main findings including the strength of evidence for each main outcome; consider their relevance to key groups (e.g., healthcare providers, users, and policy makers). | Discussion, paragraph 1-6 | |
| Limitations | | 25 | | Discuss limitations at study and outcome level (e.g., risk of bias), and at review-level (e.g., incomplete retrieval of identified research, reporting bias). | Discussion, paragraph 6 | |
| **CONCLUSIONS** | | | | |  | |
| Conclusions | | 26 | | Provide a general interpretation of the results in the context of other evidence, and implications for future research. | Conclusion, paragraph 1 | |
| **FUNDING** | | | | |  | |
| Funding | | 27 | | Describe sources of funding for the systematic review and other support (e.g., supply of data); role of funders for the systematic review. | Funding | |

*From:*  Moher D, Liberati A, Tetzlaff J, Altman DG, The PRISMA Group (2009). Preferred Reporting Items for Systematic Reviews and Meta-Analyses: The PRISMA Statement. PLoS Med 6(7): e1000097. doi:10.1371/journal.pmed1000097

For more information, visit: **www.prisma-statement.org**.

**Table S2** Search strategies

| Search | Search Terms |
| --- | --- |
| #1 | “blood routine examination” OR “blood cell” OR leukocyte OR neutrophil OR lymphocyte OR monocyte OR eosinophil OR basophil OR platelet |
| #2 | cytokines OR interleukin OR IL OR interferon OR IFN OR “tumor necrosis factor” OR TNF OR “colony-stimulating factor” OR CSF OR chemokine OR “tumor transforming growth factor” OR TGF OR “vascular endothelial cell growth factor” OR VEGF OR “endothelial cell growth factor” OR EGF OR “fibroblast growth factor” OR FGF OR PDGF OR CD4 OR CD8 |
| #3 | “novel coronavirus disease” OR “corona virus disease” OR COVID OR nCoV |
| #4 | (#1 OR #2) AND #3 |

| **Table S3** Characteristics of the articles included in our meta-analysis | | | | | | | |
| --- | --- | --- | --- | --- | --- | --- | --- |
| **Author** | **Research time** | **Region** | **Sample size (N/M/F)** | **Age**  **(years)** | **Clinical types and case number** | **Immune inflammatory parameters** | **AHRQ score** |
| Zhe Zhu[[1](#_ENREF_1)] | 2020.1.23-2.20 | Ningbo, Zhejiang | 127/45/82 | 50.90±15.26 | Severe: 16  Non-severe: 111 | WBC, NEUT, LYM, PLT, IL-2, IL-4, IL-6, IL-10, IFN-γ, TNF-α | 8 |
| Yaqing Zhou[[2](#_ENREF_2)] | 2020.1.28-3.2 | Huangshi, Hubei | 21/13/8 | 66.10±13.94 | Severe: 8  Critical: 13 | WBC, NEUT, LYM, PLT, HB, EOS, BAS, MONO, RBC | 7 |
| Bo Diao[[3](#_ENREF_3)] | 2019.12-2020.1 | Wuhan, Hubei | 522/-/- | NA | Non-ICU care: 479  ICU care: 20 | CD4^+^, CD8^+^ | 7 |
| Guang Chen[[4](#_ENREF_4)] | To 2020.2.2 | Wuhan, Hubei | 21/17/4 | 56 (50, 65) | Severe: 11  Moderate: 10 | CD4^+^, CD8^+^, CD16^+^CD56^+^ | 7 |
| Xiaohua Chen[[5](#_ENREF_5)] | 2020.2.1-2.19 | Wuhan, Hubei | 48/31/17 | 64.6±18.1 | Moderate: 21  Severe: 10  Critical: 17 | WBC, NEUT, LYM, IL-6 | 8 |
| Yong Gao[[6](#_ENREF_6)] | 2020.1.23-2.2 | Fuyang, Anhui | 43/26/17 | 43.74±12.12 | Severe: 15  Mild: 28 | WBC, LYM, MONO, IL-6 | 7 |
| Ruyuan He[[7](#_ENREF_7)] | 2020.1.10-2.13 | Wuhan, Hubei | 204/79/125 | 49 (34, 62) | Severe: 69  Non-severe: 135 | WBC, NEUT, LYM, PLT, CD3^+^, CD4^+^, CD8^+^, CD19^+^, CD16^+^CD56^+^, IL-2, IL-4, IL-5, IL-6, IL-10, IFN-γ, TNF-α | 9 |
| Chaolin Huang[[8](#_ENREF_8)] | From 2020.1.2 | Wuhan, Hubei | 41/30/11 | 49 (41, 58) | Non-ICU care: 28  ICU care: 13 | WBC, NEUT, LYM, PLT, HB | 8 |
| Jing Liu[[9](#_ENREF_9)] | 2020.1.5-1.24 | Wuhan, Hubei | 40/15/25 | 48.7±13.9 | Severe: 13  Mild: 27 | WBC, NEUT, LYM, HB, MONO | 8 |
| Yang Liu[[10](#_ENREF_10)] | 2020.1-2020.2 | Nanchang, Jiangxi | 76/49/27 | 45 | Severe: 30  Mild: 46 | LYM, CD4^+^, CD8^+^, IL-1b, IL-2R, IL-6, IL-8, IL-10 | 9 |
| Rong Qu[[11](#_ENREF_11)] | 2020.1-2020.2 | Huizhou, Guangdong | 30/16/14 | 50.5 (36, 65) | Severe: 3  Non-severe: 27 | LYM, PLT | 7 |
| Suyu Sun[[12](#_ENREF_12)] | 2020.1.19-2.20 | Wenzhou, Zhejiang | 116/60/56 | 50 (41, 57) | Common: 89  Severe: 27 | WBC, NEUT, LYM, PLT, HB, EOS, MONO | 9 |
| Suxin Wan[[13](#_ENREF_13)] | 2020.1.23-2.8 | Chongqing | 135/72/63 | 47 (36, 55) | Severe: 40  Mild: 95 | WBC, NEUT, LYM | 9 |
| Feng Wang[[14](#_ENREF_14)] | 2020.1 | Wuhan, Hubei | 65/37/28 | 57.11±13.03 | Mild: 30  Severe: 20  Extremely severe: 15 | WBC, NEUT, LYM | 9 |
| Xiaoying Xia[[15](#_ENREF_15)] | 2020.1.23-2.18 | Wuhan, Hubei | 10/6/4 | 56.5±11.16 | Ordinary: 7  Severe: 3 | WBC, LYM, CD4^+^ | 6 |
| Fang Zheng[[16](#_ENREF_16)] | 2020.1.17-2.7 | Changsha, Hunan | 161/80/81 | 45 (33.5, 57) | Severe: 30  Non-severe: 131 | WBC, LYM, PLT, HB | 8 |
| Jinjin Zhang[[17](#_ENREF_17)] | 2020.1.16-2.3 | Wuhan, Hubei | 140/71/69 | 57 (25-87)* | Severe: 56  Non-severe: 82 | WBC, LYM, EOS | 8 |
| Guqin Zhang[[18](#_ENREF_18)] | 2020.1.2-2.10 | Wuhan, Hubei | 221/108/113 | 55.0 (39.0, 66.5) | Severe: 55  Non-severe: 166 | WBC, NEUT, LYM, PLT, MONO | 9 |
| Qingchun Yao[[19](#_ENREF_19)] | 2020.2.2-3.3 | Huanggang, Hubei | 108/43/65 | 52 (37, 58) | Severe: 25  Non-severe: 83 | WBC, NEUT, LYM, PLT, HB | 8 |
| Bo Xu[[20](#_ENREF_20)] | 2019.12.26-2020.3.1 | Wuhan, Hubei | 187/103/84 | 62.0 (48.5, 71.0) | Mild: 80  Severe: 45  Critical: 62 | WBC, NEUT, LYM, MONO, CD3^+^, CD4^+^, CD8^+^, CD16^+^CD56^+^, IL-1b, IL-6, IL-10 | 9 |
| Hansheng Xie[[21](#_ENREF_21)] | 2020.1-2020.2 | Wuhan, Hubei | 79/44/35 | 60.0 (48.0, 66.0) | Severe: 28  Moderate: 51 | WBC, NEUT, LYM | 8 |
| Zhongyong Chang[[22](#_ENREF_22)] | 2020.1-2020.2 | Wuhan, Hubei | 150/80/70 | 55.27±6.33 | Mild: 48  Ordinary: 45  Severe: 35  Critical: 22 | IL-6 | 8 |
| Lei Chen[[23](#_ENREF_23)] | 2020.1.14-1.29 | Wuhan, Hubei | 29/21/8 | 56 | Ordinary: 15  Severe: 9  Critical: 5 | LYM, IL-1b, IL-2R, IL-6, IL-8, IL-10, TNF-α | 6 |
| Min Chen[[24](#_ENREF_24)] | 2020.1.24-2020.2.8 | Wuhan, Hubei | 54/27/27 | 58.5 (43.8, 69) | Ordinary: 23  Severe: 25 | WBC, NEUT, LYM, HB | 7 |
| Shi Chen[[25](#_ENREF_25)] | 2019.12.24-2020.1.28 | Wuhan, Hubei | 109/48/61 | 52.5±10.8 | Ordinary: 65  Severe: 44 | WBC, LYM | 8 |
| Wen Chen[[26](#_ENREF_26)] | To 2020 .2.21 | Jingmen, Hubei | 91/45/46 | 41.59±15.53 | Mild: 2  Ordinary: 68  Severe: 12  Critical: 9 | WBC, LYM | 9 |
| Xi Chen[[27](#_ENREF_27)] | 2020.1-2020.2 | Chongqing | 139/76/63 | 46 (36, 54) | Mild: 108  Severe: 31 | WBC, NEUT, LYM | 9 |
| Xing Chen[[28](#_ENREF_28)] | 2020.1.27-2.15 | Guangzhou, Guangdong | 296/137/159 | (15-87) * | Ordinary: 266  Severe: 30 | WBC, NEUT, LYM, PLT | 7 |
| Kebin Cheng[[29](#_ENREF_29)] | To 2020.2.6 | Wuhan, Hubei | 463/244/219 | 51 (43, 60) | Ordinary: 282  Critical: 181 | WBC, LYM, PLT, HB, IL-6 | 9 |
| Xiaowei Fang[[30](#_ENREF_30)] | 2020.1.22-2.8 | Hefei, Anhui | 79/45/34 | 45.1±16.6 | Ordinary: 55  Severe: 24 | WBC, NEUT, LYM | 7 |
| Yu Lei[[31](#_ENREF_31)] | 2020.1.10-1.30 | Wuhan, Hubei | 51/25/26 | 55 (26-82)* | Ordinary: 32  Severe: 9  Critical: 10 | WBC, NEUT, LYM | 8 |
| Dan Li a[[32](#_ENREF_32)] | 2020.1.22-2.8 | Shenyang, Liaoning | 30/18/12 | 43 (21-72)* | Mild/Ordinary: 20  Severe: 9  Critical: 1 | WBC, NEUT, LYM, PLT, HB | 7 |
| Dan Li b[[33](#_ENREF_33)] | 2020.1.20-2.27 | Zhuzhou, Hunan | 80/40/40 | 47.8±19.5 | Mild/Ordinary: 63  Severe: 11  Critical: 6 | WBC, LYM, PLT, HB | 8 |
| Dan Li c[[34](#_ENREF_34)] | 2020.1.31-2.25 | Wuhan, Hubei | 62/32/30 | 43 (29-75)  54.5 (37-70)  68 (42-85) | Ordinary: 18  Severe: 22  Critical: 22 | WBC, NEUT, LYM, PLT, CD3^+^, CD4^+^, CD8^+^, CD19^+^, CD16^+^CD56^+^ | 8 |
| Min Liu[[35](#_ENREF_35)] | 2020.1.10-1.31 | Wuhan, Hubei | 30/10/20 | 35±8 | Ordinary: 26  Severe: 4 | WBC, LYM | 7 |
| Sijia Liu[[36](#_ENREF_36)] | 2020.1.23-2.12 | Ezhou, Hubei | 342/183/159 | 56 (45, 67) | Ordinary: 196  Severe: 97  Critical: 49 | WBC, PLT | 9 |
| Yingxia Liu[[37](#_ENREF_37)] | 2020.1.11-1.20 | Shenzhen, Guangdong | 12/8/4 | 53.67±18.01 | Ordinary: 4  Severe: 5  Critical: 3 | WBC, NEUT, LYM, PLT, CD4^+^, CD8^+^ | 6 |
| Yudong Peng[[38](#_ENREF_38)] | 2020.1.20-2.15 | Wuhan, Hubei | 112/53/59 | 62 (55, 67) | Ordinary:96  Critical: 16 | WBC, NEUT, LYM, MONO | 8 |
| Xia Song[[39](#_ENREF_39)] | 2020.1.25-2.21 | Lanzhou, Gansu | 28/17/11 | 39.73 (1.8-74) | Ordinary: 19  Critical: 9 | IL-6 | 6 |
| Jingsong Tang[[40](#_ENREF_40)] | 2020.1-2020.2 | Dongguan, Guangdong | 40/26/14 | 15-79* | Mild: 6  Ordinary: 23  Severe: 8  Critical: 3 | IL-6 | 7 |
| Qiu Wan[[41](#_ENREF_41)] | 2020.1.26-2.5 | Chongqing | 153/77/76 | 45.36±3.13 | Mild/Ordinary: 132  Severe: 21 | LYM, CD3^+^, CD4^+^, CD8^+^ | 9 |
| Tianxin Xiang[[42](#_ENREF_42)] | 20201.21-1.27 | Nanchang, Jiangxi | 49/33/16 | 42.9 (18-78)* | Ordinary:40  Severe/Critical:9 | WBC, NEUT, LYM, PLT, MONO, HB, EOS, CD3^+^, CD4^+^, CD8^+^, CD19^+^, CD16^+^CD56^+^ | 7 |
| Kaihu Xiao[[43](#_ENREF_43)] | 2020.1.23-2.8 | Chongqing | 143/73/70 | 45.13±1.04 | Ordinary: 107  Severe: 24  Critical: 12 | WBC, LYM, PLT, IL-6 | 8 |
| Juan Xiong[[44](#_ENREF_44)] | 2020.1.17-2.20 | Wuhan, Hubei | 89/41/49 | 53.0±16.9 | Mild: 18  Ordinary: 40  Severe: 21  Critical: 10 | WBC, NEUT, LYM, PLT, MONO, HB | 7 |
| Jing Xu[[45](#_ENREF_45)] | 2020.1.12-2.18 | Fuyang, Anhui | 155/87/68 | 41.99±15.40 | Ordinary: 125  Severe: 30 | CD4^+^, CD8^+^, IL-6 | 8 |
| Jing Yuan[[46](#_ENREF_46)] | 2020.1.24-2.23 | Chongqing | 223/106/117 | 46.5±16.1 | Ordinary: 192  Severe: 31 | WBC, LYM, HB | 9 |
| Tingting Zhan[[47](#_ENREF_47)] | 2020.1.20-2.20 | Guangzhou, Guangdong | 40/23/17 | 60 (25-90)* | Ordinary: 13  Severe: 16  Critical: 11 | WBC, NEUT, LYM, PLT, EOS | 7 |
| Fangtian Zuo[[48](#_ENREF_48)] | 2020.1.19-3.20 | Nanyang, Henan | 50/18/32 | 48.2±15.3 | Ordinary: 39  Severe: 11 | WBC, LYM, PLT | 7 |
| Qingxu Zou[[49](#_ENREF_49)] | 2020.2-2020.3 | Wuhan, Hubei | 50/23/27 | 66.9±10.73 | Ordinary: 7  Severe: 35  Critical: 8 | IL-6, TNF-α, IL-2R | 8 |
| Shaohua Zhong[[50](#_ENREF_50)] | 2020.1.21-2.10 | Haikou, Hainan | 62/40/22 | 51.8±13.5 | Ordinary: 51  Severe: 2  Critical: 9 | WBC, NEUT, LYM | 8 |
| Wei Zhang[[51](#_ENREF_51)] | 2020.1.21-2.11 | Beijing | 74/35/39 | 52.7±19.1 | Ordinary: 56  Severe: 9  Critical: 9 | WBC, NEUT, LYM, PLT, HB | 7 |
| Yanfei Bin[[52](#_ENREF_52)] | 2020.1.29-2.16 | Wuhan, Hubei | 55/31/24 | 53.9±17.1 | Mild: 45  Severe: 9 | WBC, LYM | 8 |
| Ning Guo[[53](#_ENREF_53)] | 2020.1-2020.2 | Wuhan, Hubei | 122/62/60 | 54 (23-86)* | Mild/Ordinary: 92  Severe/Critical: 30 | WBC, LYM | 8 |
| Yaxiong Hunag[[54](#_ENREF_54)] | 2020.1.30-2.20 | Changsha, Hunan | 121/64/57 | 45.13±13.89 | Mild/Ordinary: 92  Severe/Critical: 29 | WBC, HB, PLT, EOS | 8 |
| Guohua Li[[55](#_ENREF_55)] | 2020.1.29-3.3 | Wuhan, Hubei | 110/56/54 | 63.91±11.77 | Ordinary: 57  Severe: 37  Critical: 16 | IL-6, IL-8, IL-10, CD4^+^, CD8^+^ | 9 |
| Ruiyun Li[[56](#_ENREF_56)] | 2020.1.31-2.14 | Wuhan, Hubei | 110/67/43 | 64.6±13.5 | Ordinary: 9  Severe: 62  Critical: 39 | WBC | 8 |
| Ruoqing Li[[57](#_ENREF_57)] | 2020.1.21-3.2 | Xiaogan,  Hubei | 193/112/81 | 50.7±16.2 | Ordinary: 122  Severe: 71 | WBC, NEUT, LYM, PLT, HB | 9 |
| Yaling Shi[[58](#_ENREF_58)] | 2020.1-2020.2 | Guangzhou,  Guangdong | 160/73/87 | 49±15.55 | Ordinary: 150  Severe: 8  Critical: 6 | WBC, LYM | 8 |
| Dagang Wang[[59](#_ENREF_59)] | 2020.2-2020.3 | Beijing | 77/43/34 | 47.98±21.02 | Ordinary: 49  Severe: 28 | LYM | 7 |
| Zhiwei Xie[[60](#_ENREF_60)] | 2020.1.20-3.2 | Guangzhou,  Guangdong | 280/130/150 | 49.5 (35.0, 62.0) | Mild:225  Severe:55 | WBC, NEUT, LYM, PLT, HB | 9 |
| Mengyao Ji[[61](#_ENREF_61)] | 2020.1.2-1.28 | Wuhan, Hubei | 101/48/53 | 51.0 (37.0, 61.0) | Mild: 88  Severe: 55  Critical: 14 | WBC, NEUT, LYM, PLT, MONO | 8 |
| Yongpo Jiang[[62](#_ENREF_62)] | 2020.1.31-2.16 | Taizhou,  Zhejiang | 60/35/25 | 41 (12, 74)* | Severe: 8  Non-severe: 52 | WBC, HB, PLT | 7 |
| Shaoqing Lei[[63](#_ENREF_63)] | 2020.1.1-2.5 | Wuhan, Hubei | 34/14/20 | 55 (43-63) | Non-ICU care: 19  ICU care: 15 | WBC, NEUT, LYM, PLT, MONO | 7 |
| Zhihua Lv[[64](#_ENREF_64)] | 2020.2.4-2.28 | Wuhan, Hubei | 354/175/179 | 62 (23-90)* | Mild: 115  Severe: 155  Critical: 84 | WBC, NEUT, LYM, IL-6, IL-10, TNF-α | 9 |
| Ruirui Wang[[65](#_ENREF_65)] | 2020.1.20-2.9 | Fuyang, Anhui | 125/54/71 | 38.76±13.80 | Mild/Ordinary: 100  Severe/Critical: 25 | WBC, NEUT, LYM, PLT, MONO, HB | 8 |
| Ping Yang[[66](#_ENREF_66)] | 2020.1-2020.3 | Chongqing | 133/72/61 | 50.81 (2, 82)* | Mild: 65  Severe: 68 | WBC, LYM, PLT, CD4^+^ | 8 |
| Qiuxiang Yang[[67](#_ENREF_67)] | 2020.1.28-2.12 | Wuhan, Hubei | 136/66/70 | 55 (44, 64) | Mild: 103  Severe/Critical: 33 | WBC, LYM, PLT | 8 |
| Ruchong Chen[[68](#_ENREF_68)] | To 2020.3.22 | China | 548/313/235 | 56.0±14.5 | Mild: 345  Severe: 155  Critical: 48 | LEU, NEUT, LYM, PLT, MONO, HB, EOS, CD3^+^, CD4^+^, CD8^+^, IL-6 | 9 |
| Xiurong Ding[[69](#_ENREF_69)] | 2020.1.21-2.17 | Beijing | 72/33/39 | 49 (37-64) | Severe: 15  Non-severe: 57 | LEU, NEUT, LYM, PLT, MONO, HB | 8 |
| Jianhong Fu[[70](#_ENREF_70)] | 2020.1.20-2.20 | Suzhou, Jiangsu | 75/45/30 | 46.6±14 | Mild: 59  Severe: 16 | WBC, NEUT, LYM, HB | 7 |
| Kyung Soo Hong[[71](#_ENREF_71)] | To 2020.3.29 | Daegu | 98/38/60 | 55.4±17.1 | Non-ICU care: 85  ICU care: 13 | WBC, NEUT, LYM, HB, PLT | 8 |

**Note:** WBC: white blood cell; RBC: red blood cell; NEUT: neutrophil; LYM: lymphocyte; PLT: platelet; HB: hemoglobin; EOS: eosinophil; BAS: basophilic cells; MONO: Monocytes cells; CD3^+^: cluster of differentiation 3^+^; CD4^+^: cluster of differentiation 4^+^; CD8^+^: cluster of differentiation 8^+^; CD19^+^: cluster of differentiation 19^+^; CD16^+^56^+^: cluster of differentiation 16^+^56^+^; IL-1β: interlcukin-1β; IL-2R: interlcukin-2R; IL-4: interlcukin-4; IL-5: interlcukin-5; IL-6: interlcukin-6; IL-8: interlcukin-8; IL-10: interlcukin-10; TNF-α: tumor necrosis factor-α; AHRQ: The Agency for Healthcare Research and Quality; In age, there are three forms, mean ±SD, median (IQR) and median (range). * stands for range: median (range) or (range). NA: not available.

**
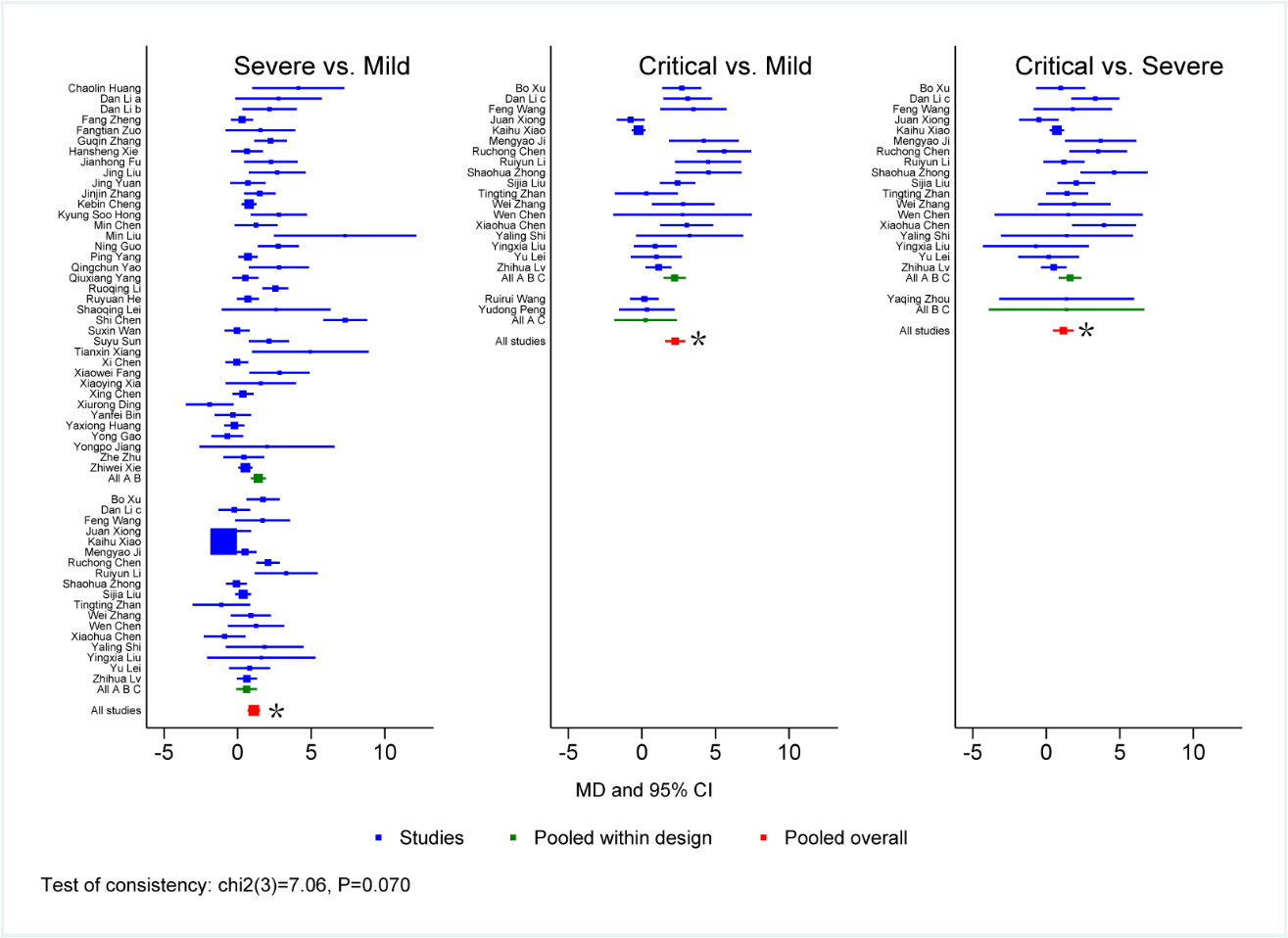
**

**Figure S1** Forest maps of white blood cell (WBC) comparison in COVID-19 patients with different clinical stages. **＊** represents statistically significant differences (*P* <0.05).


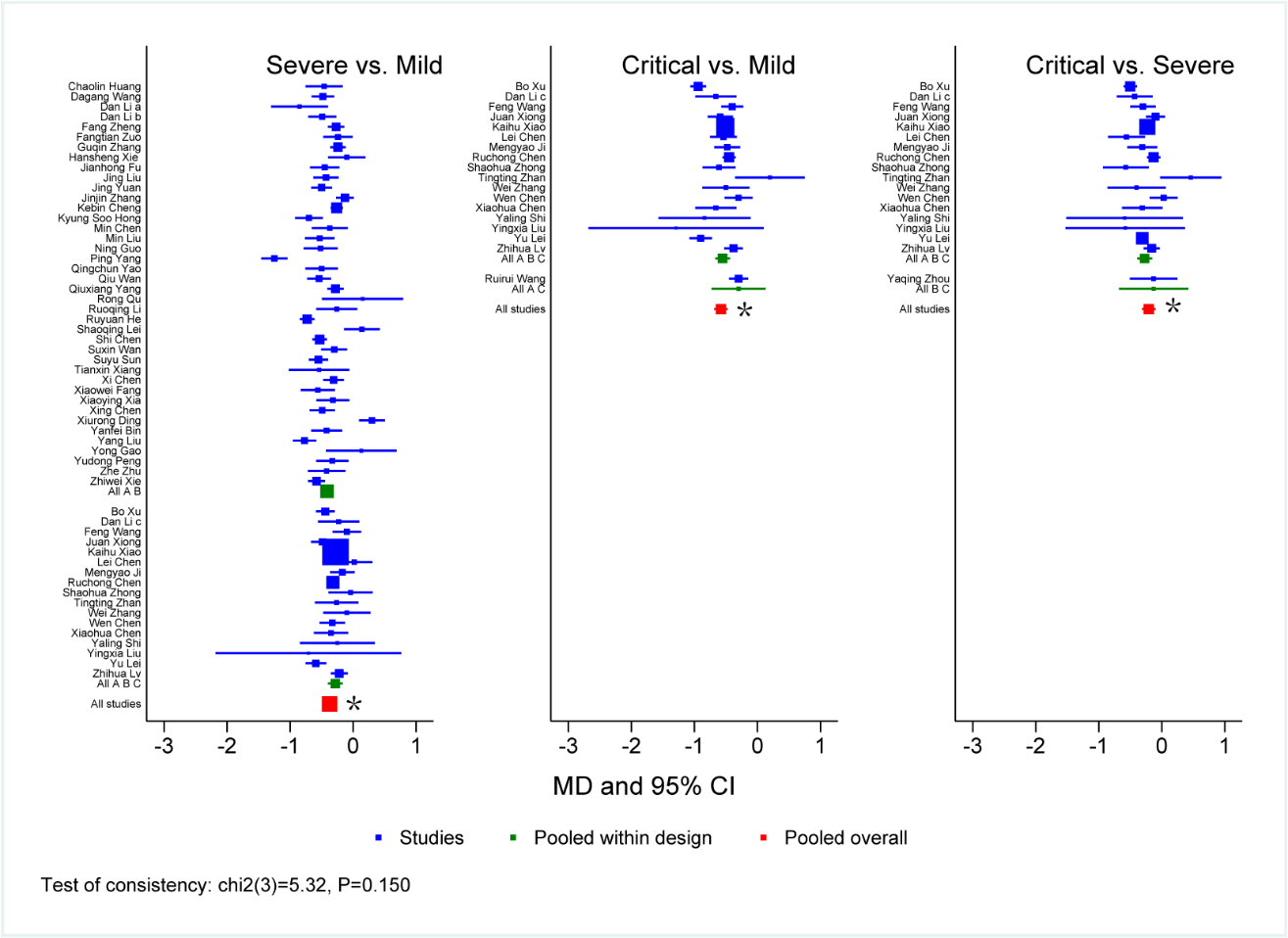


**Figure S2** Forest maps of lymphocyte (LYM) comparison in COVID-19 patients with different clinical stages. **＊** represents statistically significant differences (*P* <0.05).


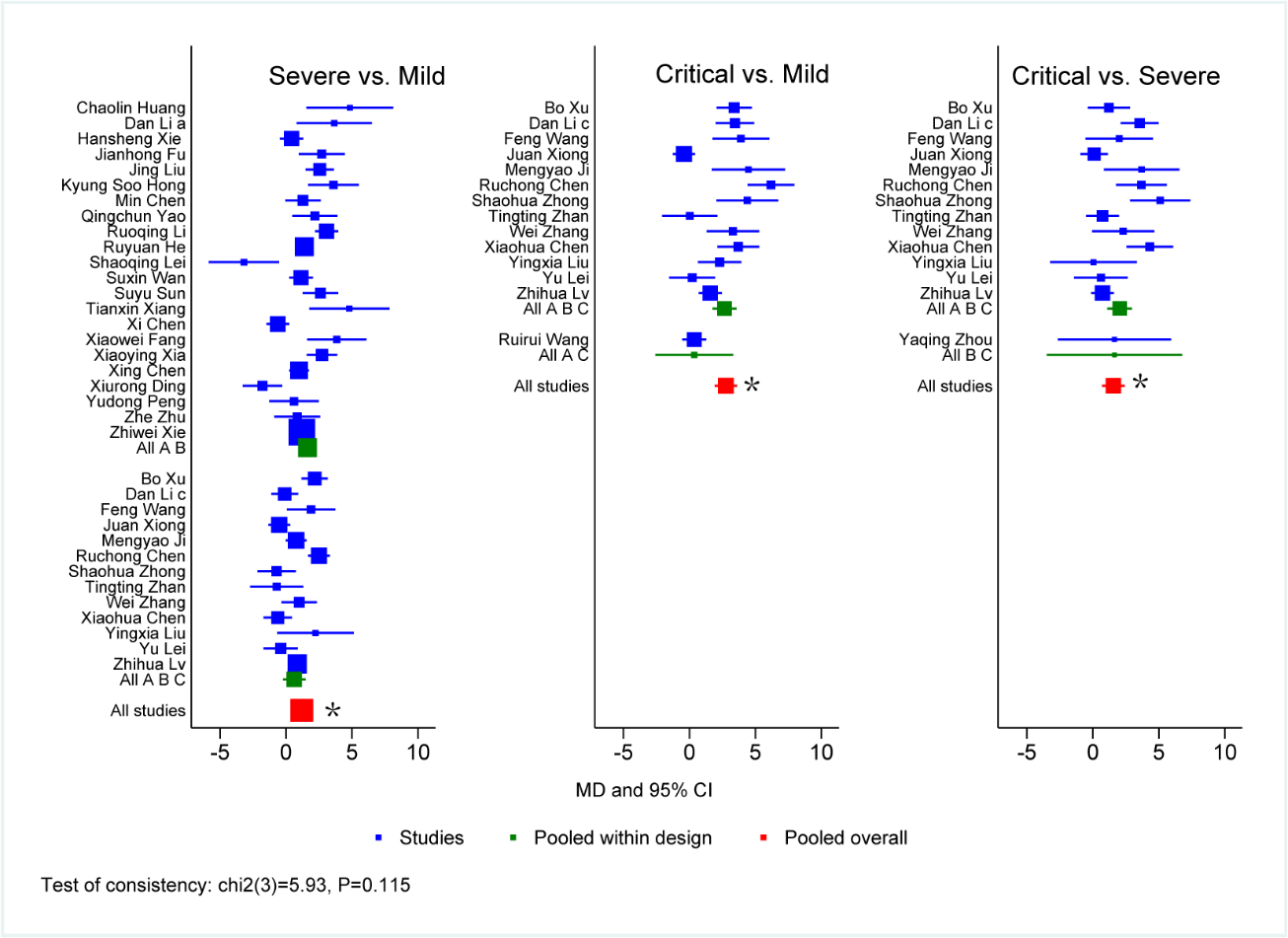


**Figure S3** Forest maps of neutrophil (NEUT) comparison in COVID-19 patients with different clinical stages. **＊** represents statistically significant differences (*P* <0.05).


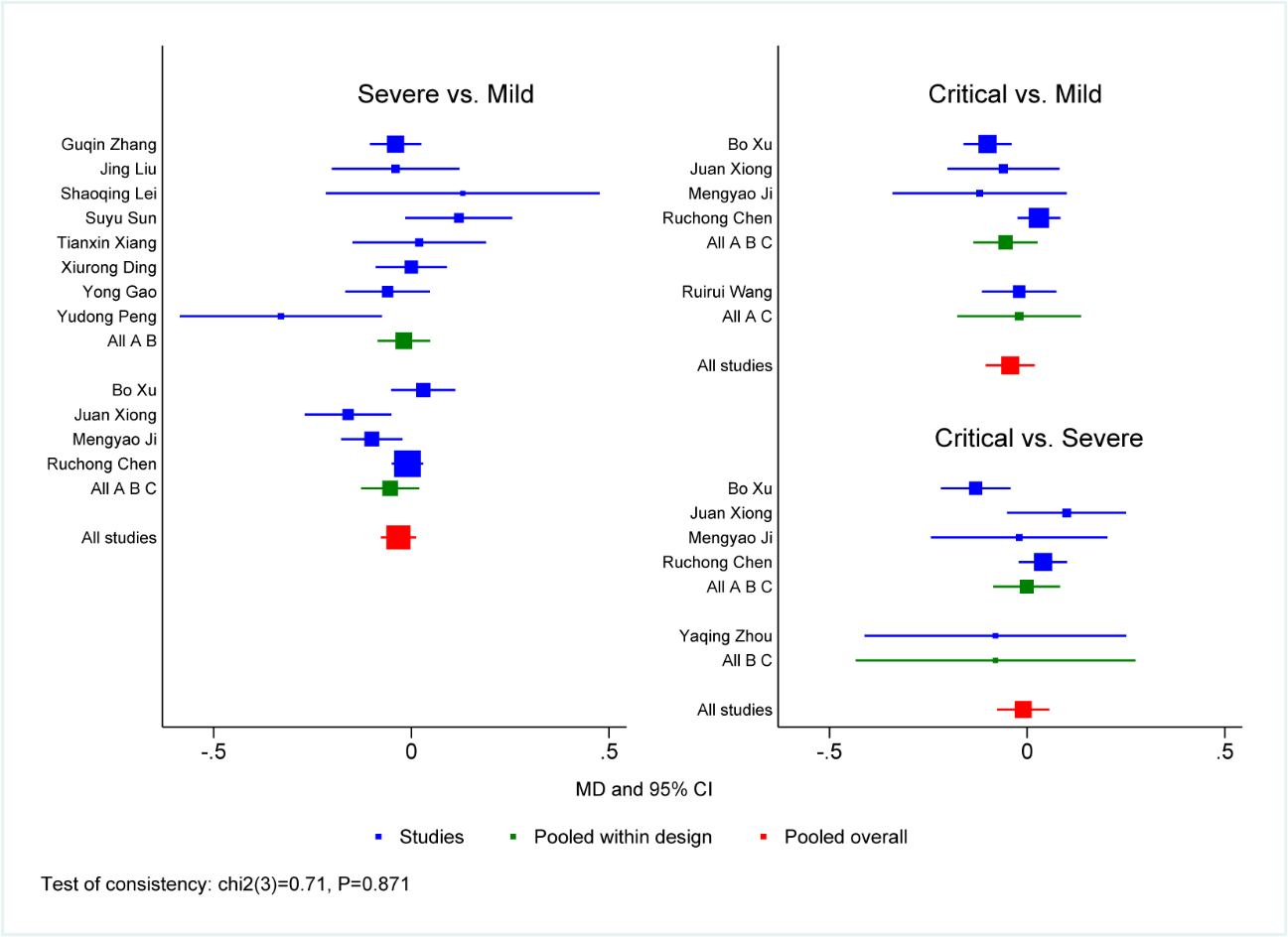


**Figure S4** Forest maps of monocytes (MONO) comparison in COVID-19 patients with different clinical stages. **＊** represents statistically significant differences (*P* <0.05).


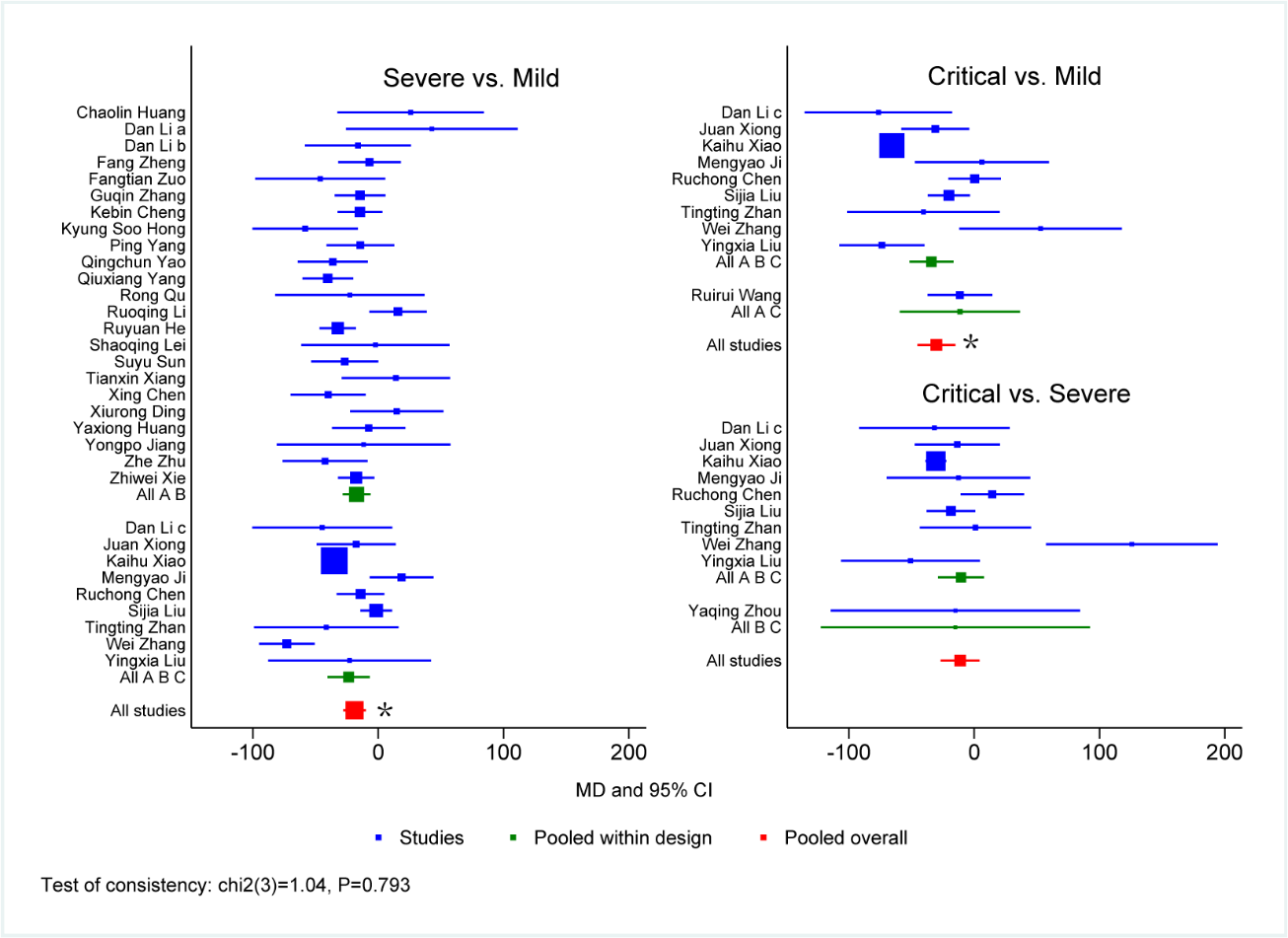


**Figure S5** Forest maps of platelet (PLT) comparison in COVID-19 patients with different clinical stages. **＊** represents statistically significant differences (*P* <0.05).


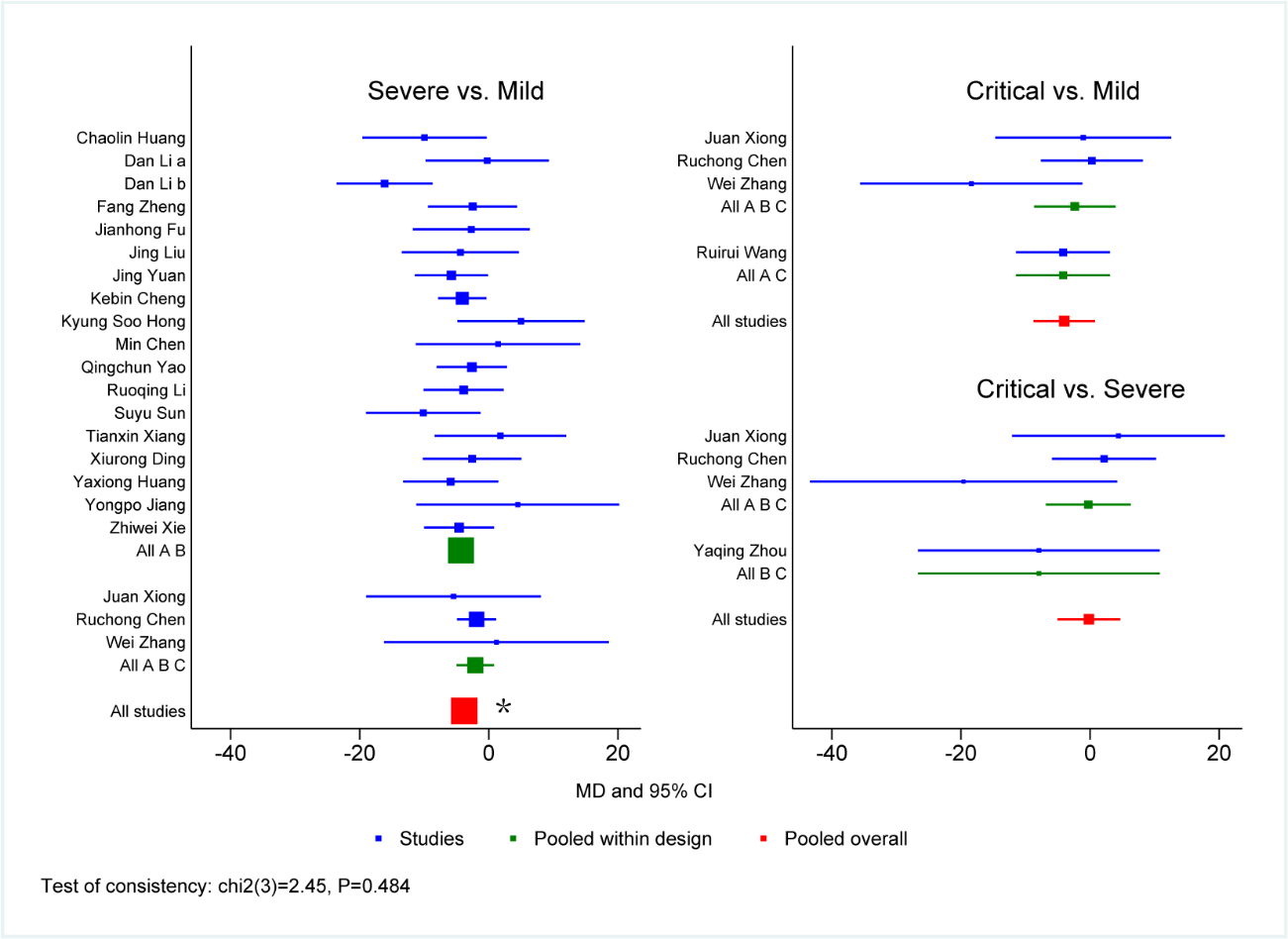


**Figure S6** Forest maps of hemoglobin (HB) comparison in COVID-19 patients with different clinical stages. **＊** represents statistically significant differences (*P* <0.05).


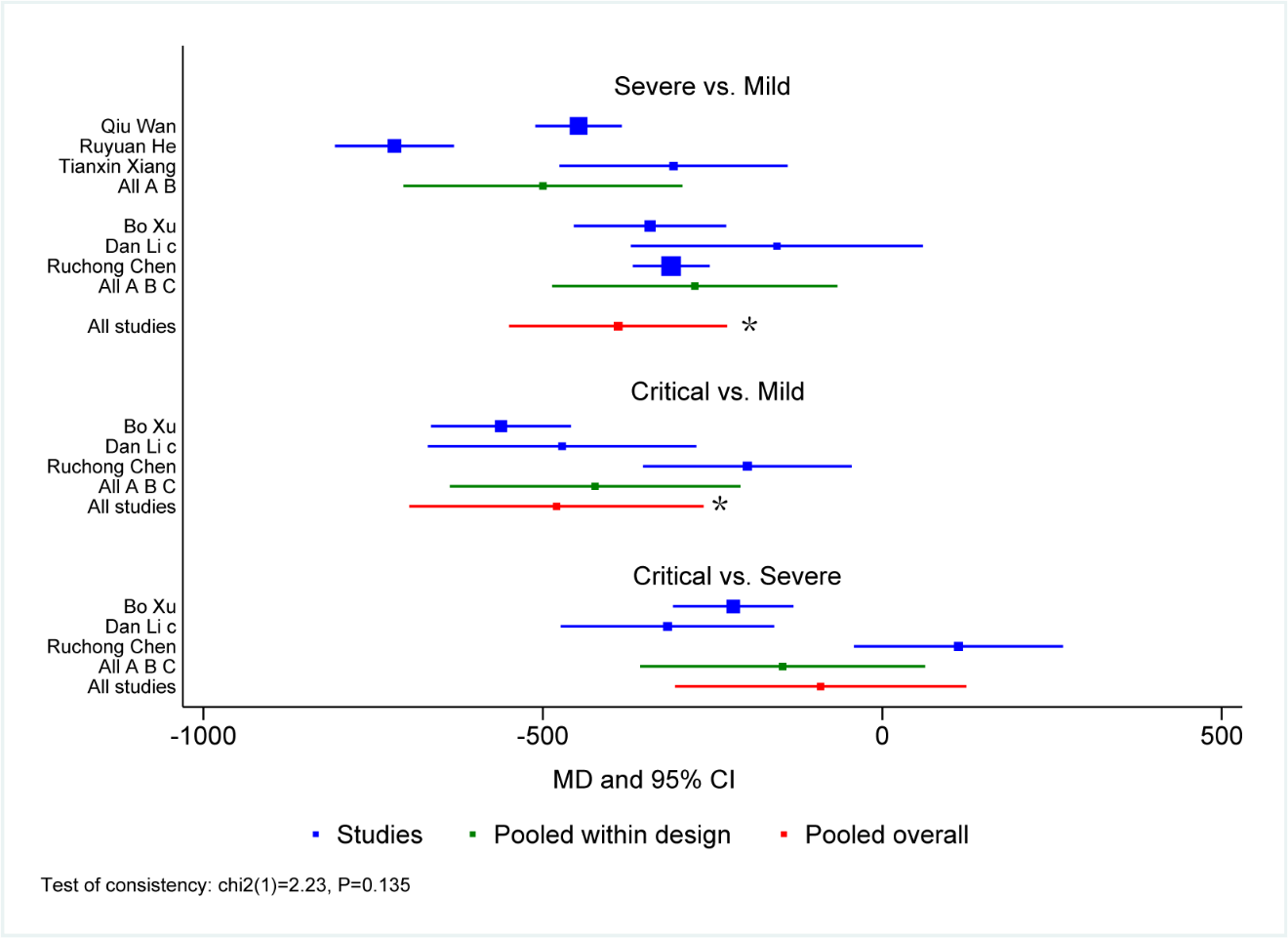


**Figure S7** Forest maps of cluster of differentiation 3 (CD3^+^) comparison in COVID-19 patients with different clinical stages. **＊** represents statistically significant differences (*P* <0.05).


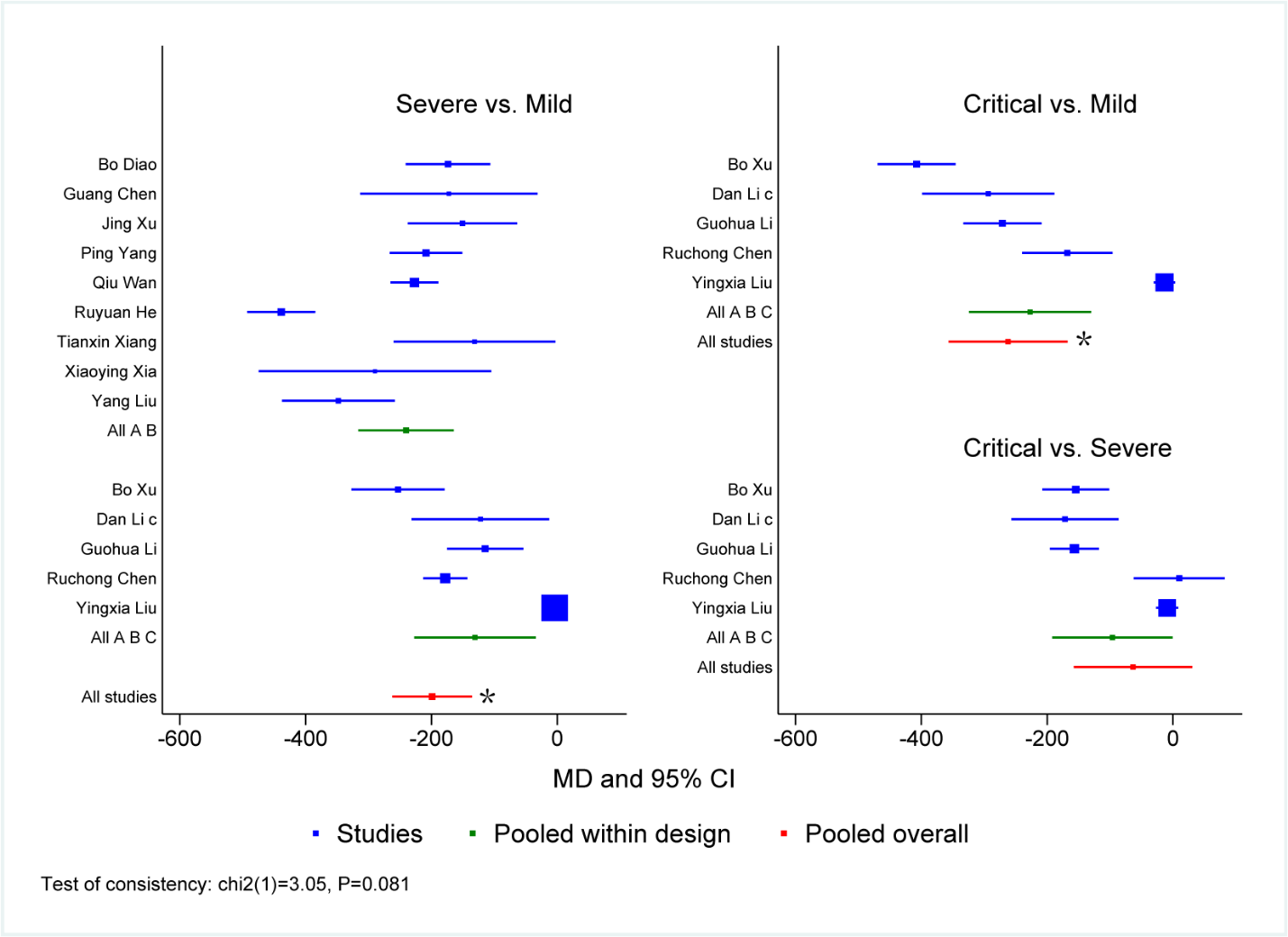


**Figure S8** Forest maps of cluster of differentiation 4 (CD4^+^) comparison in COVID-19 patients with different clinical stages: **＊**represents statistically significant differences (*P* <0.05).


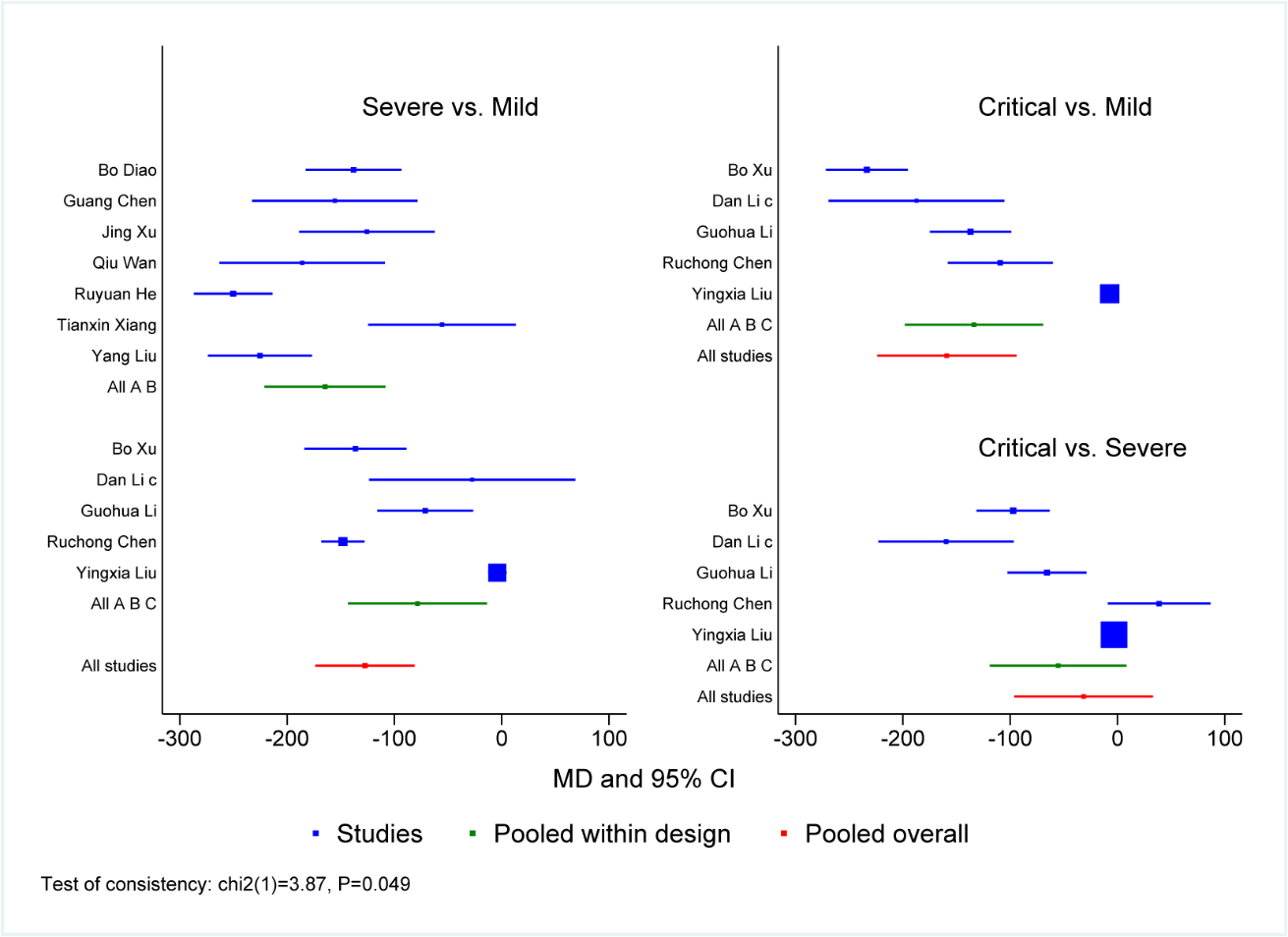


**Figure S9** Forest maps of cluster of differentiation 8 (CD8^+^) comparison in COVID-19 patients with different clinical stages: **＊**represents statistically significant differences (*P* <0.05).


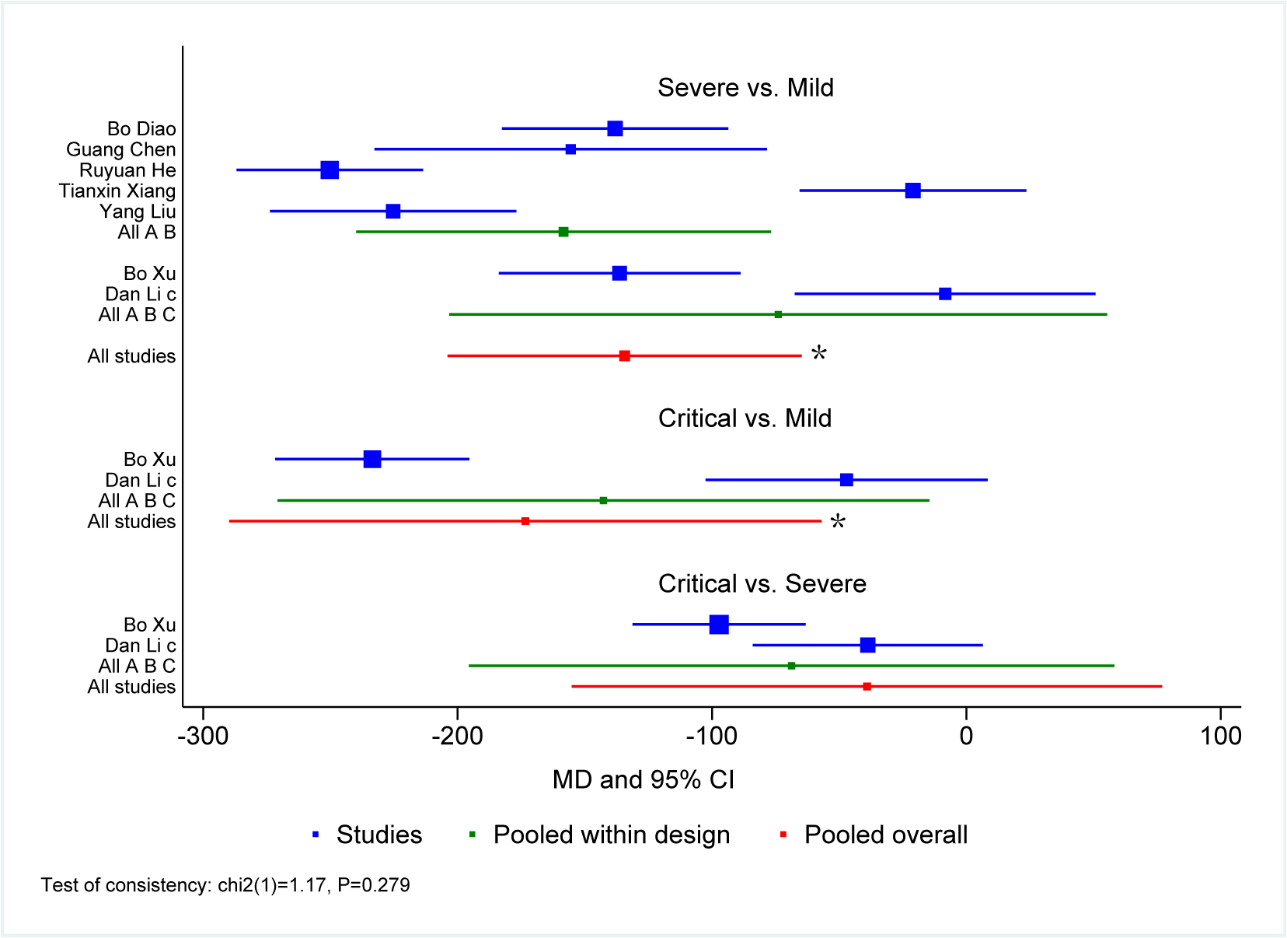


**Figure S10** Forest maps of cluster of differentiation 19 (CD19^+^) comparison in COVID-19 patients with different clinical stages: **＊**represents statistically significant differences (*P* <0.05).


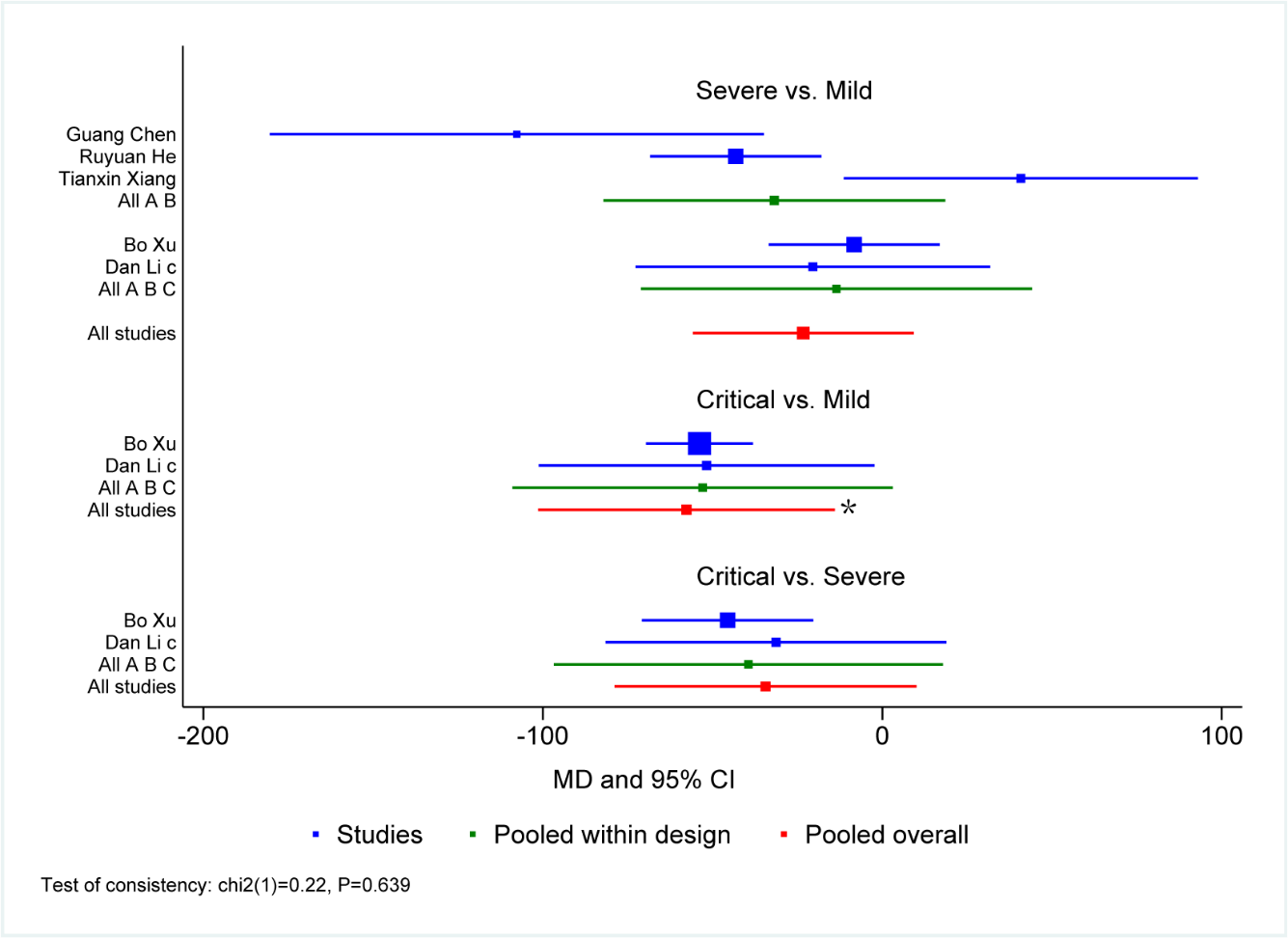


**Figure S11** Forest maps of cluster of differentiation 16^+^56^+^ (CD16^+^ CD56^+^) comparison in COVID-19 patients with different clinical stages: **＊**represents statistically significant differences (*P* <0.05).


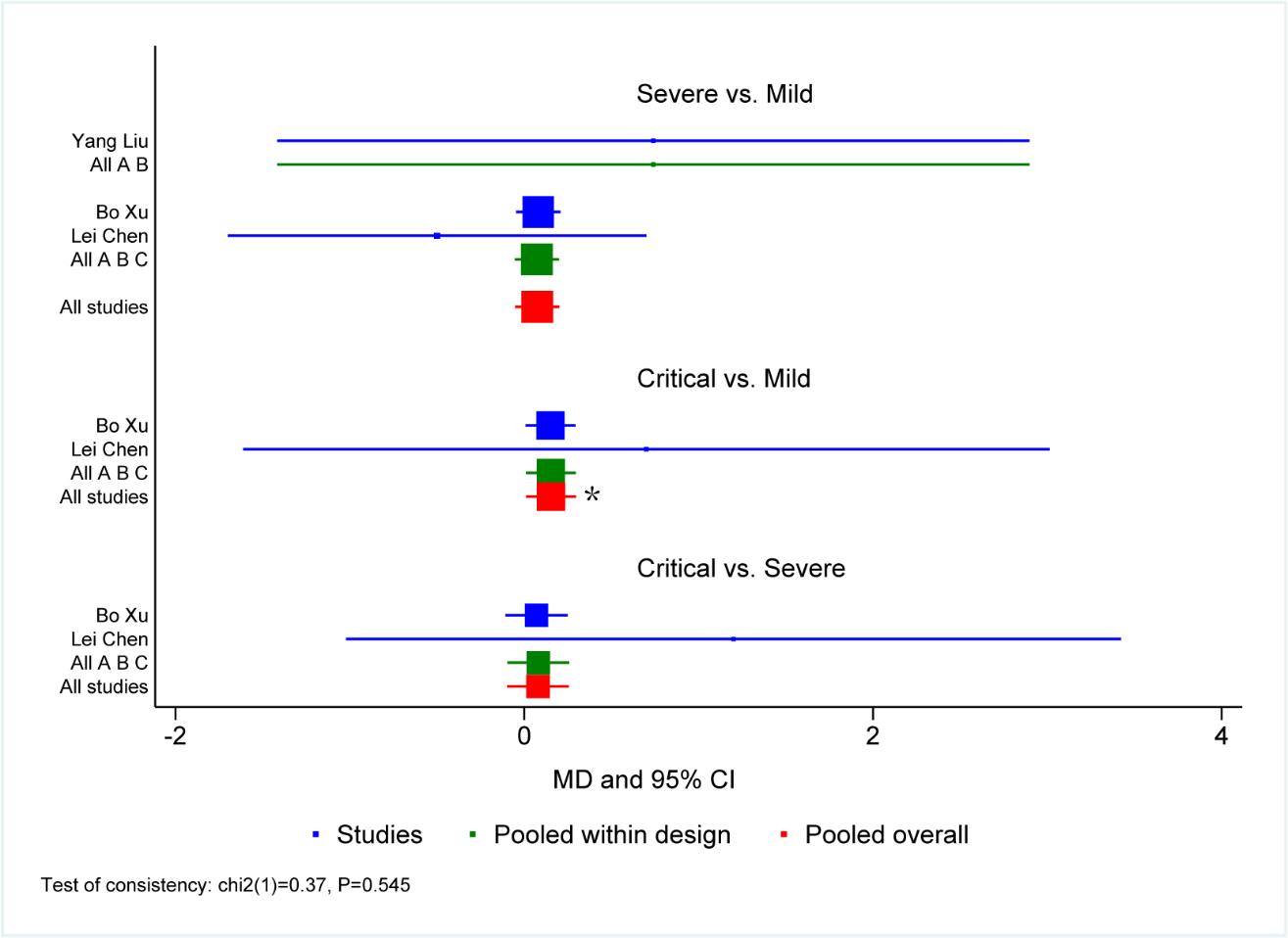


**Figure S12** Forest maps of interlcukin-1β (IL-1β) comparison in COVID-19 patients with different clinical stages. **＊** represents statistically significant differences (*P* <0.05).

**
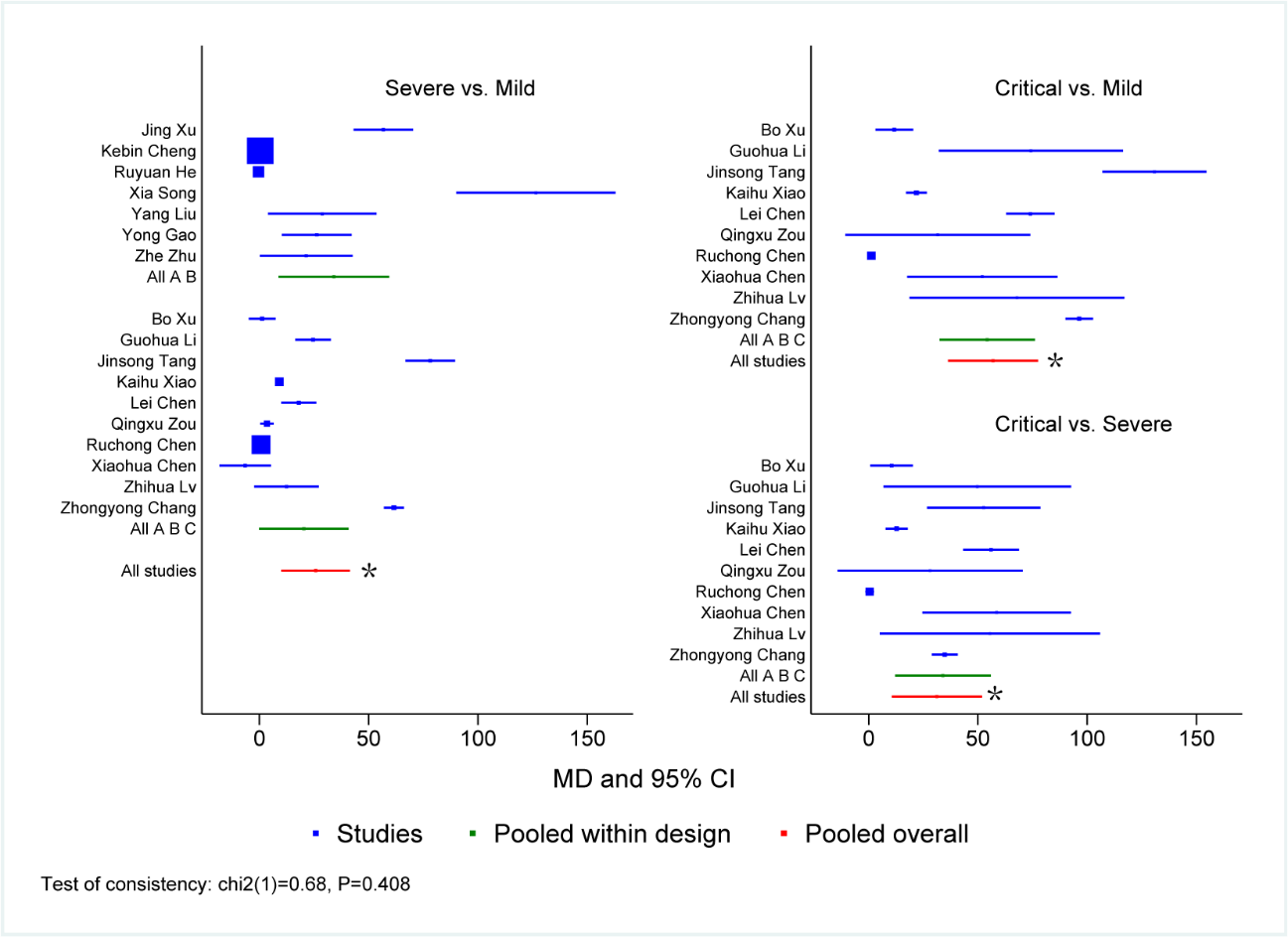
**

**Figure S13** Forest maps of interlcukin-6 (IL-6) comparison in COVID-19 patients with different clinical stages. **＊** represents statistically significant differences (*P* <0.05).

**
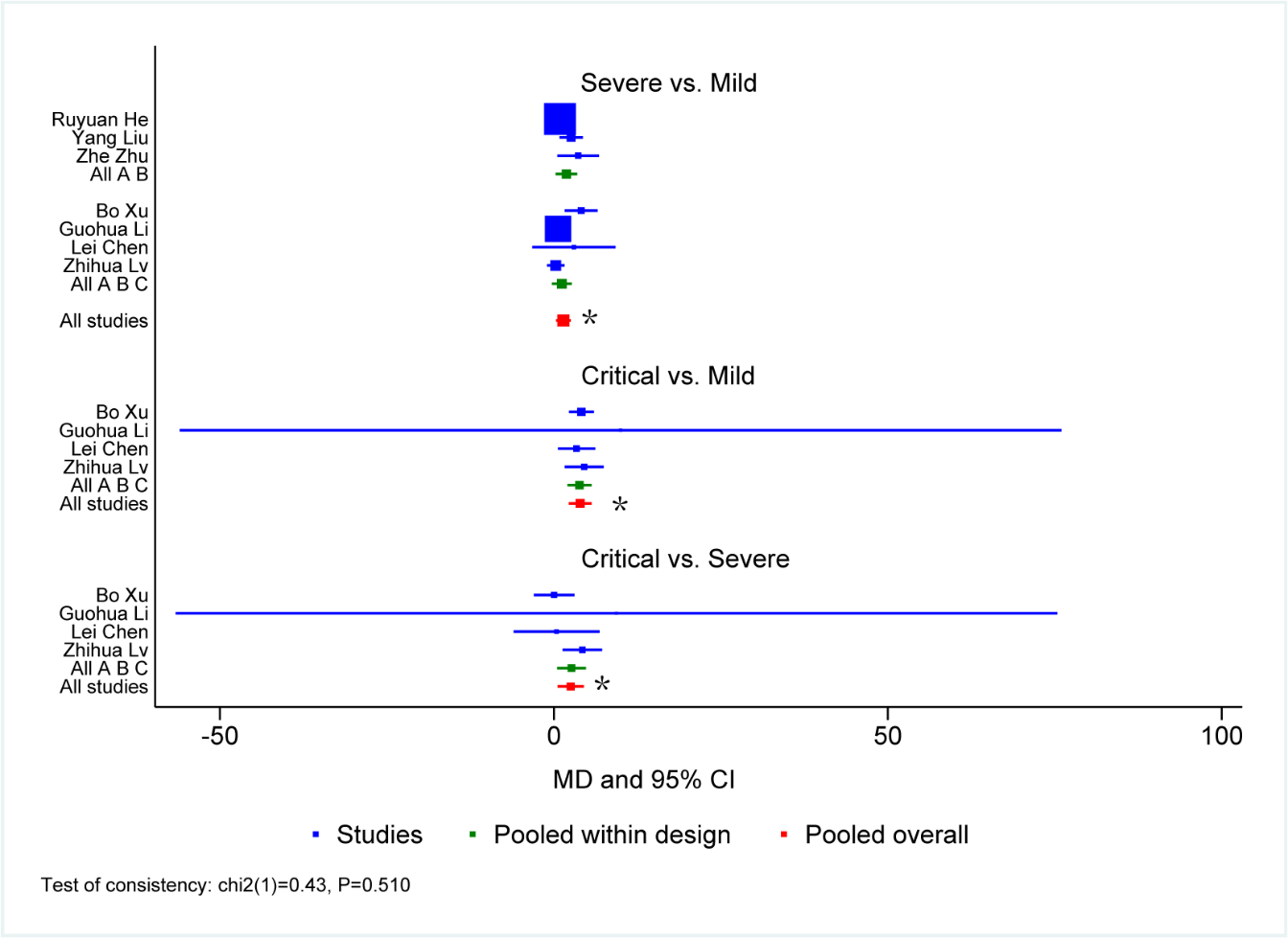
**

**Figure S14** Forest maps of interlcukin-10 (IL-10) comparison in COVID-19 patients with different clinical stages. **＊** represents statistically significant differences (*P* <0.05).

**
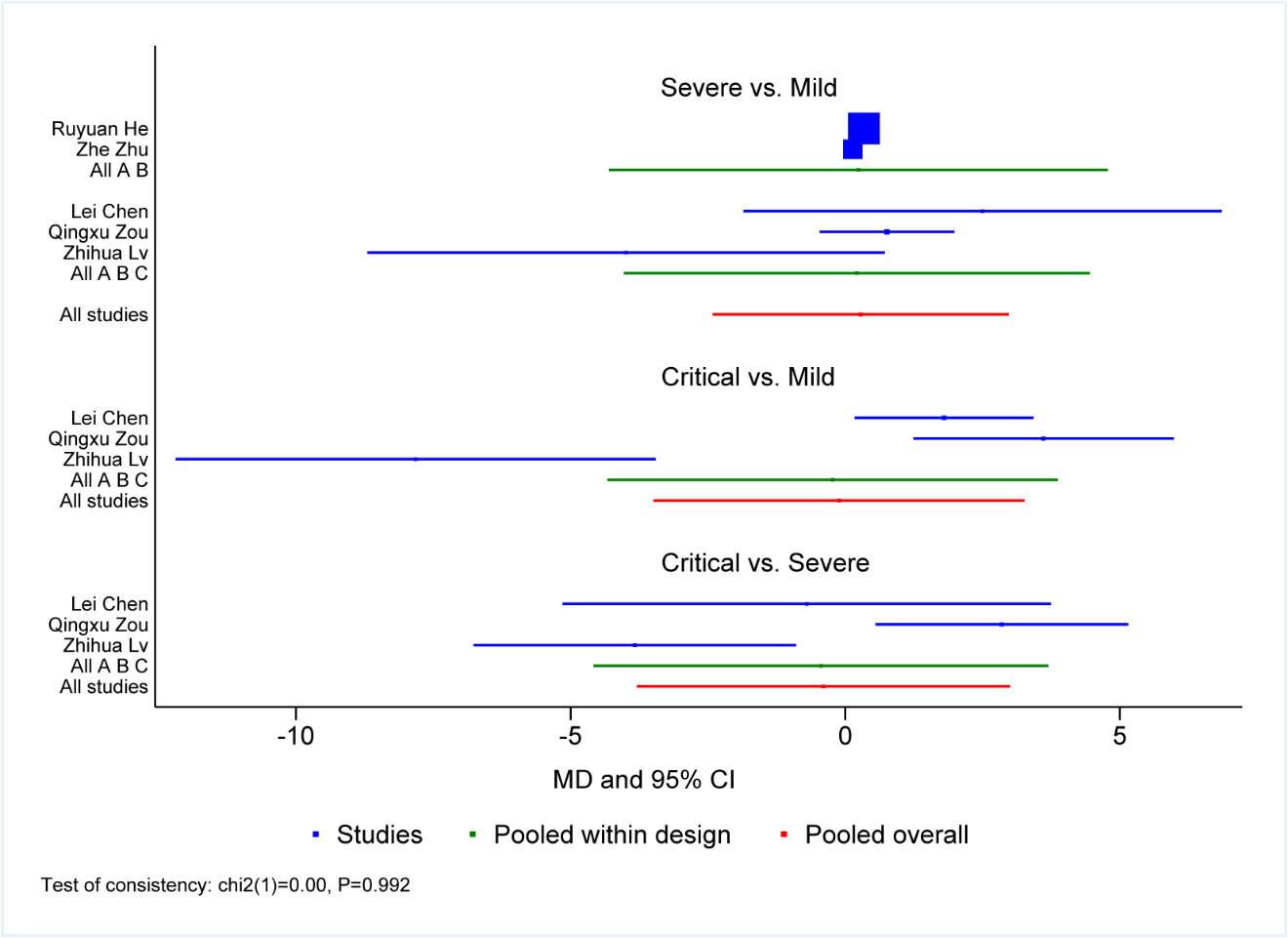
**

**Figure S15** Forest maps of tumor necrosis factor-α (TNF-α) comparison in COVID-19 patients with different clinical stages. **＊** represents statistically significant differences (*P* <0.05).

**
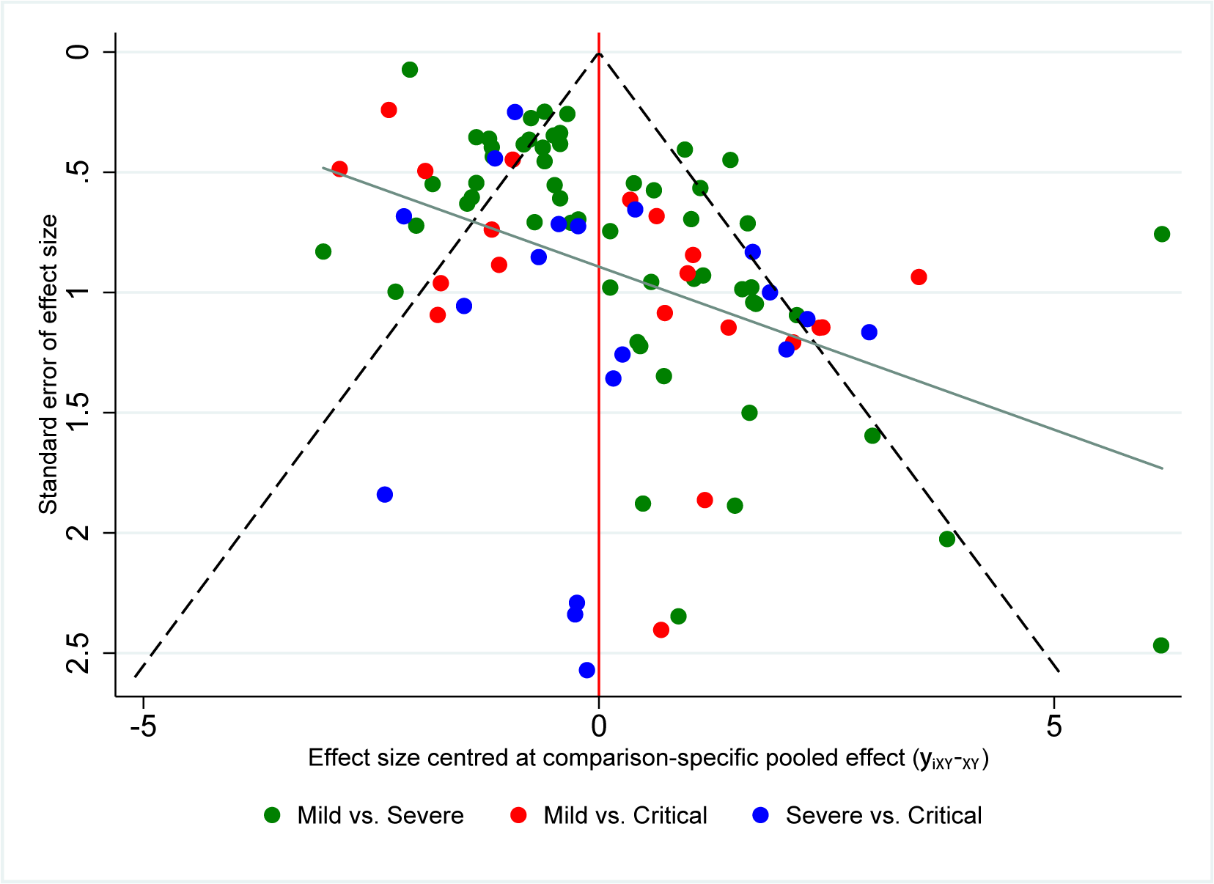
**

**Figure S16** Funnel plot, WBC

**
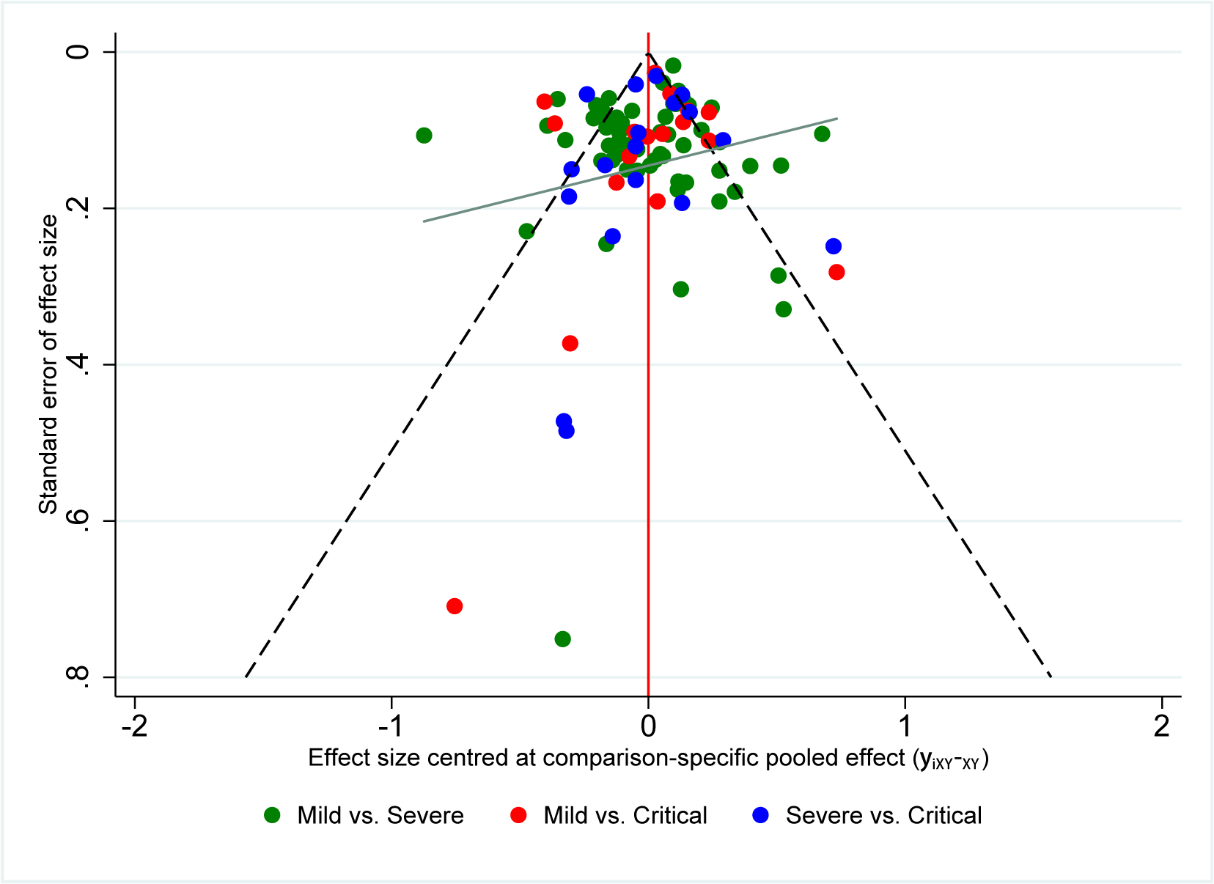
**

**Figure S17** Funnel plot, LYM

**
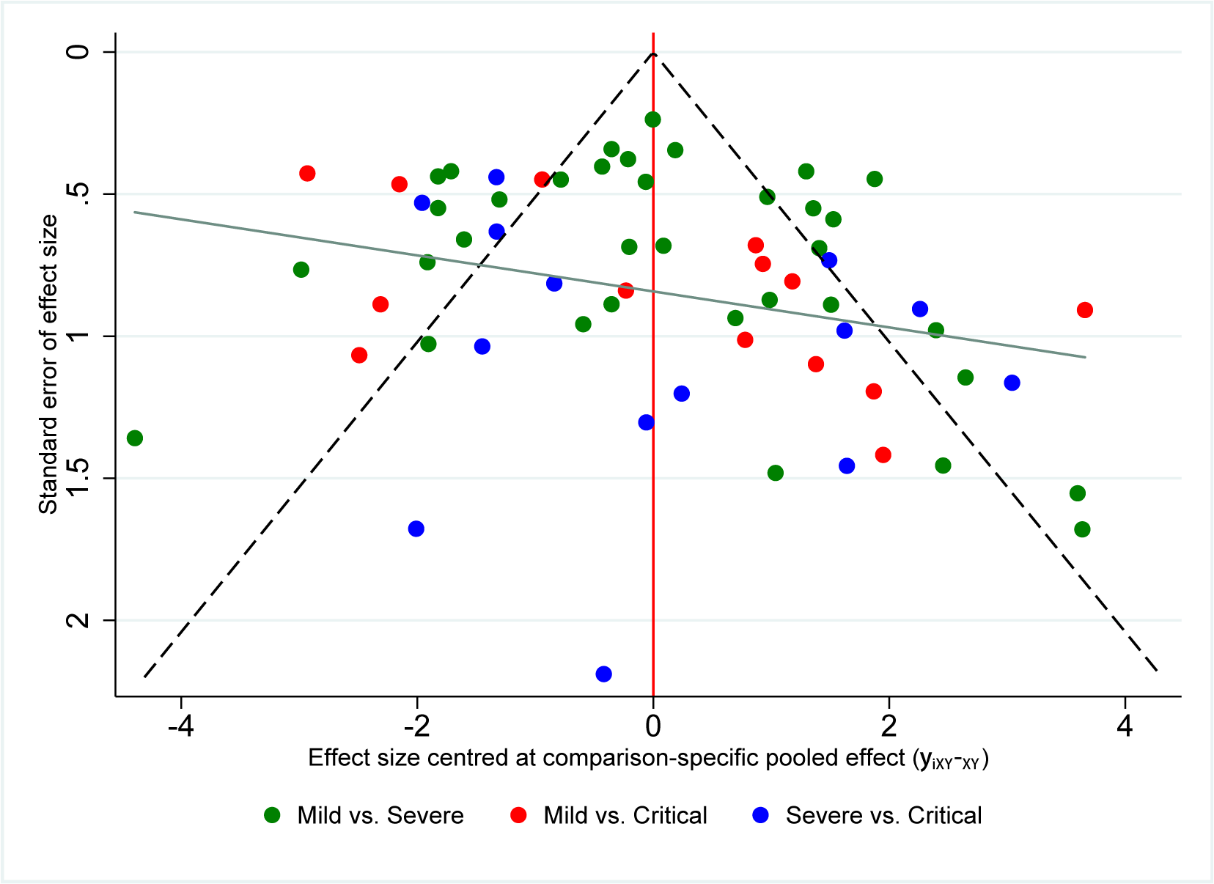
**

**Figure S18** Funnel plot, NEUT


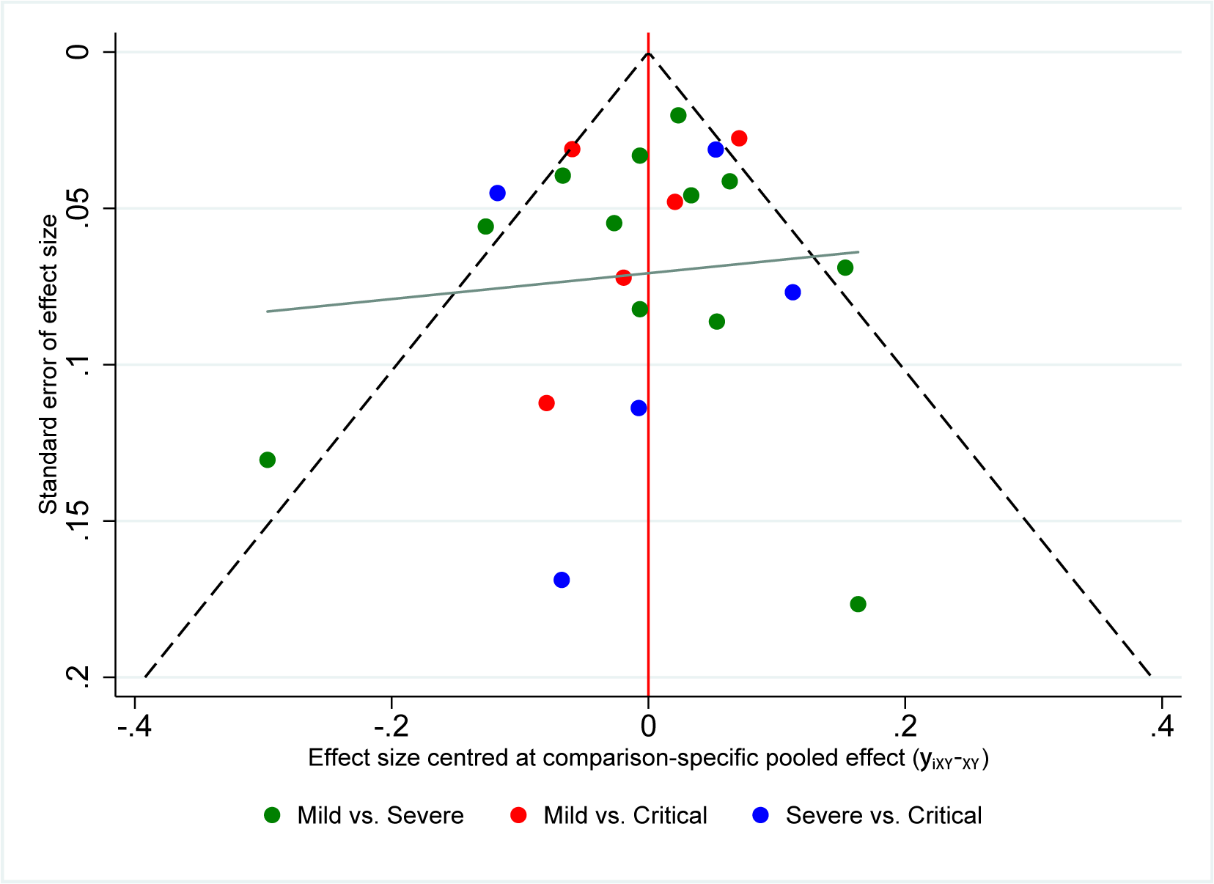


**Figure S19** Funnel plot, MONO

**
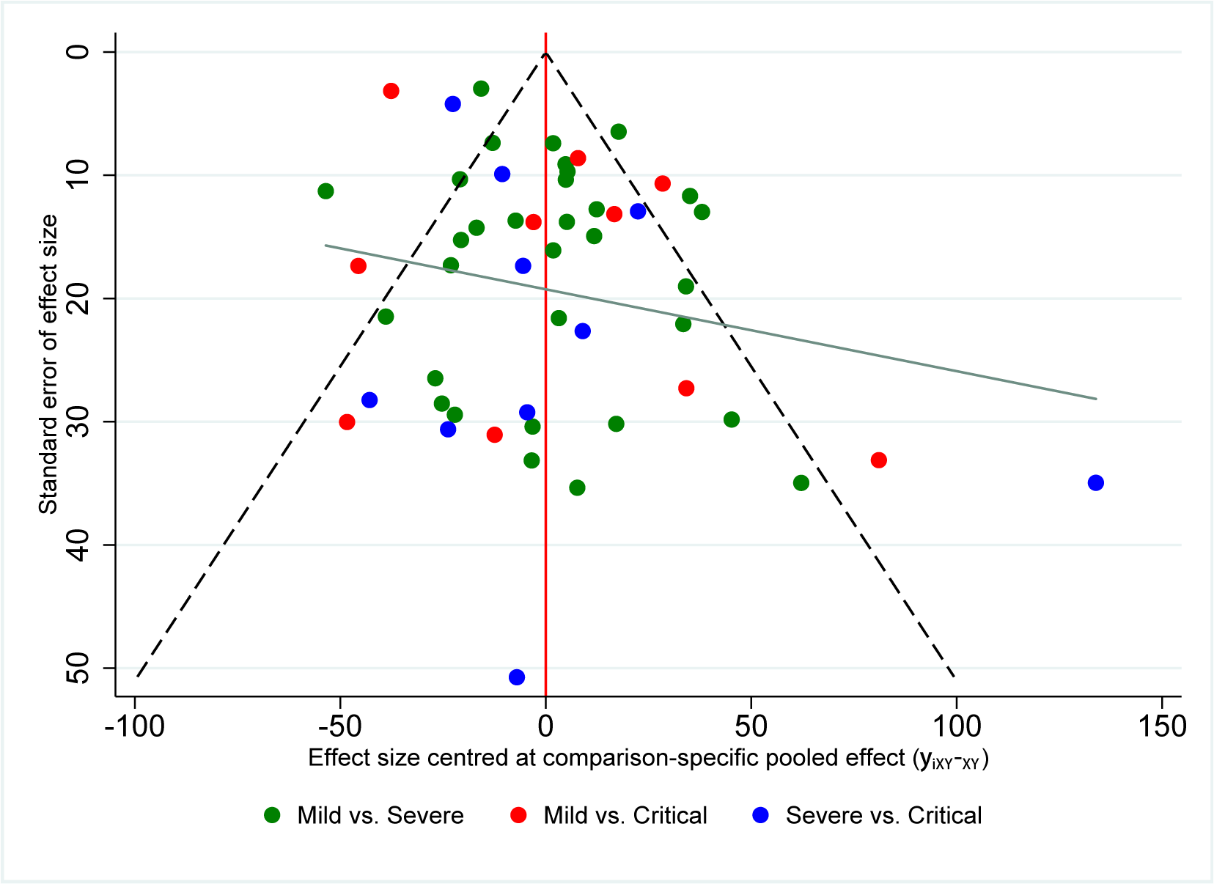
**

**Figure S20** Funnel plot, PLT

**
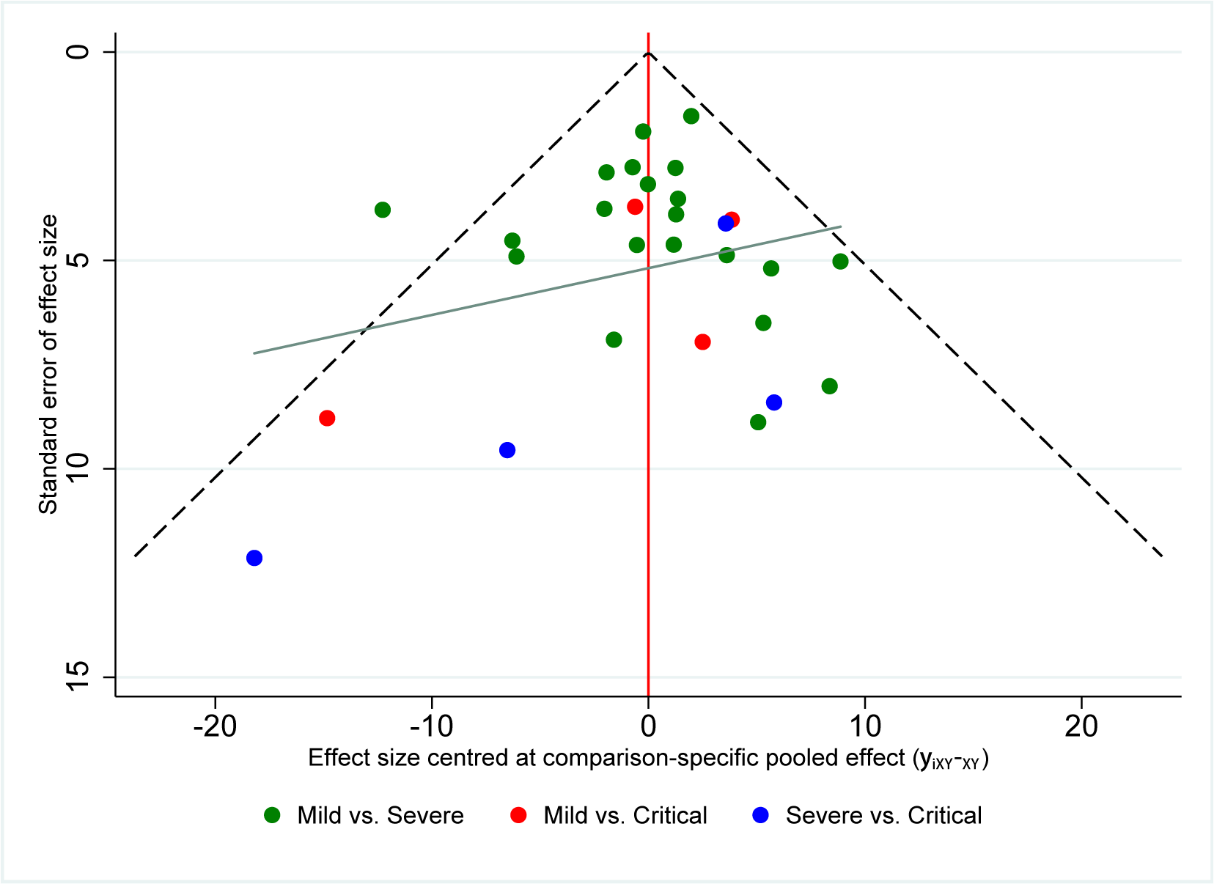
**

**Figure S21** Funnel plot, HB

**
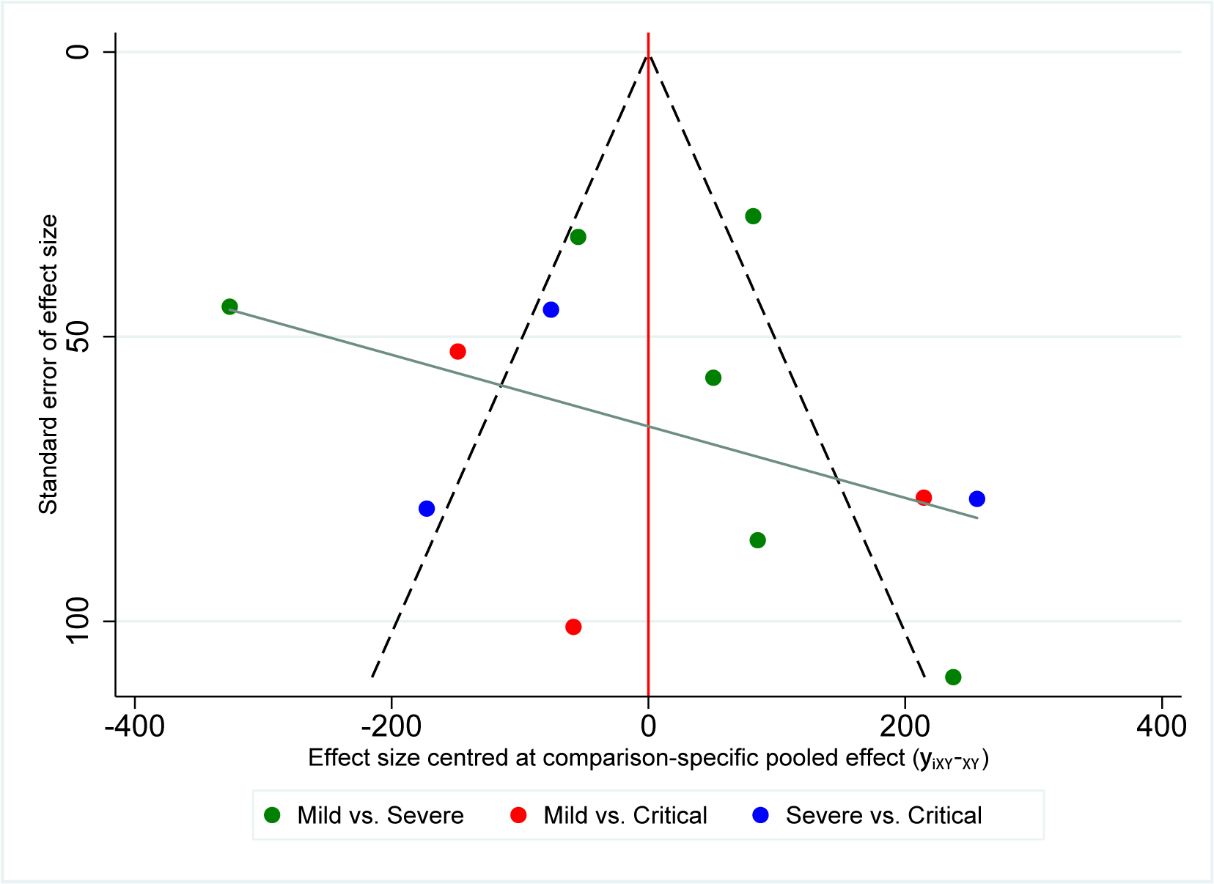
**

**Figure S22** Funnel plot, CD3^+^


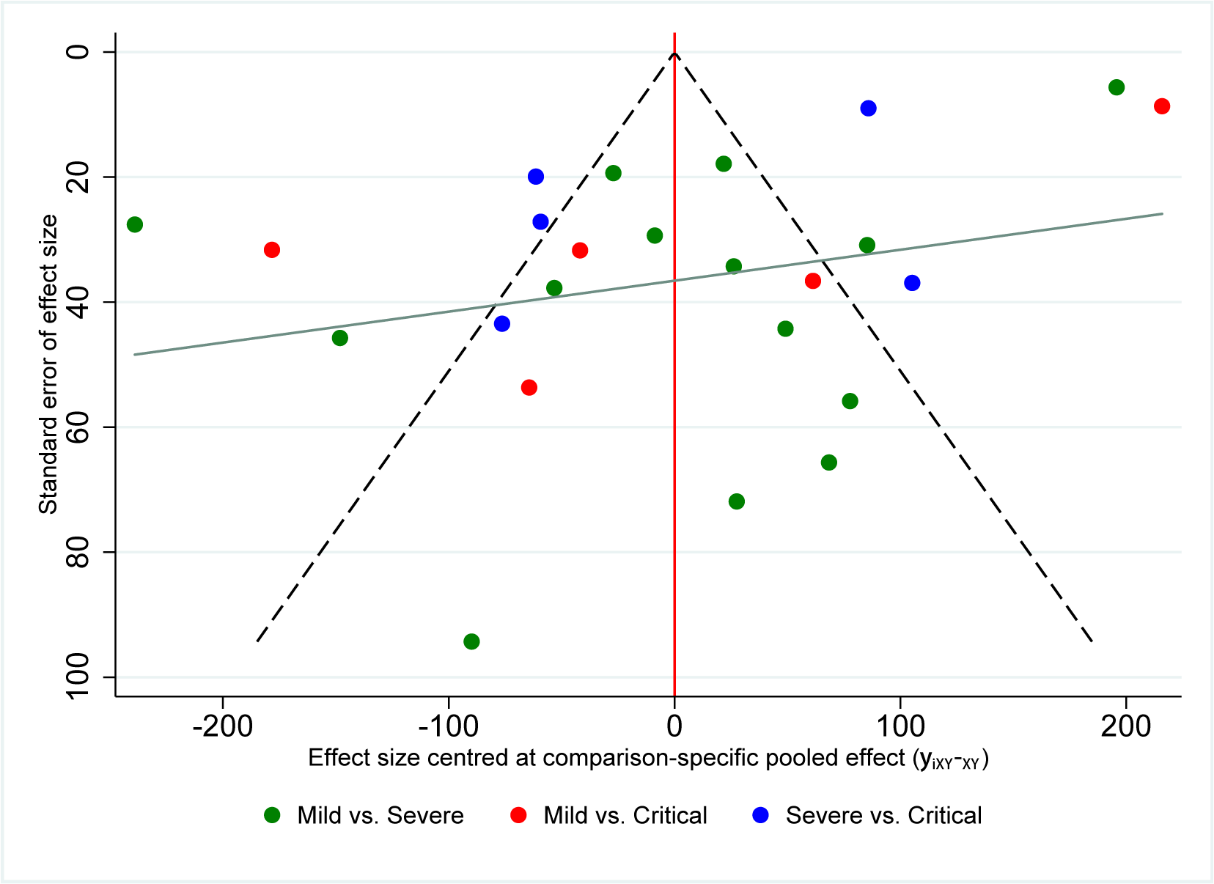


**Figure S23** Funnel plot, CD4^+^

**
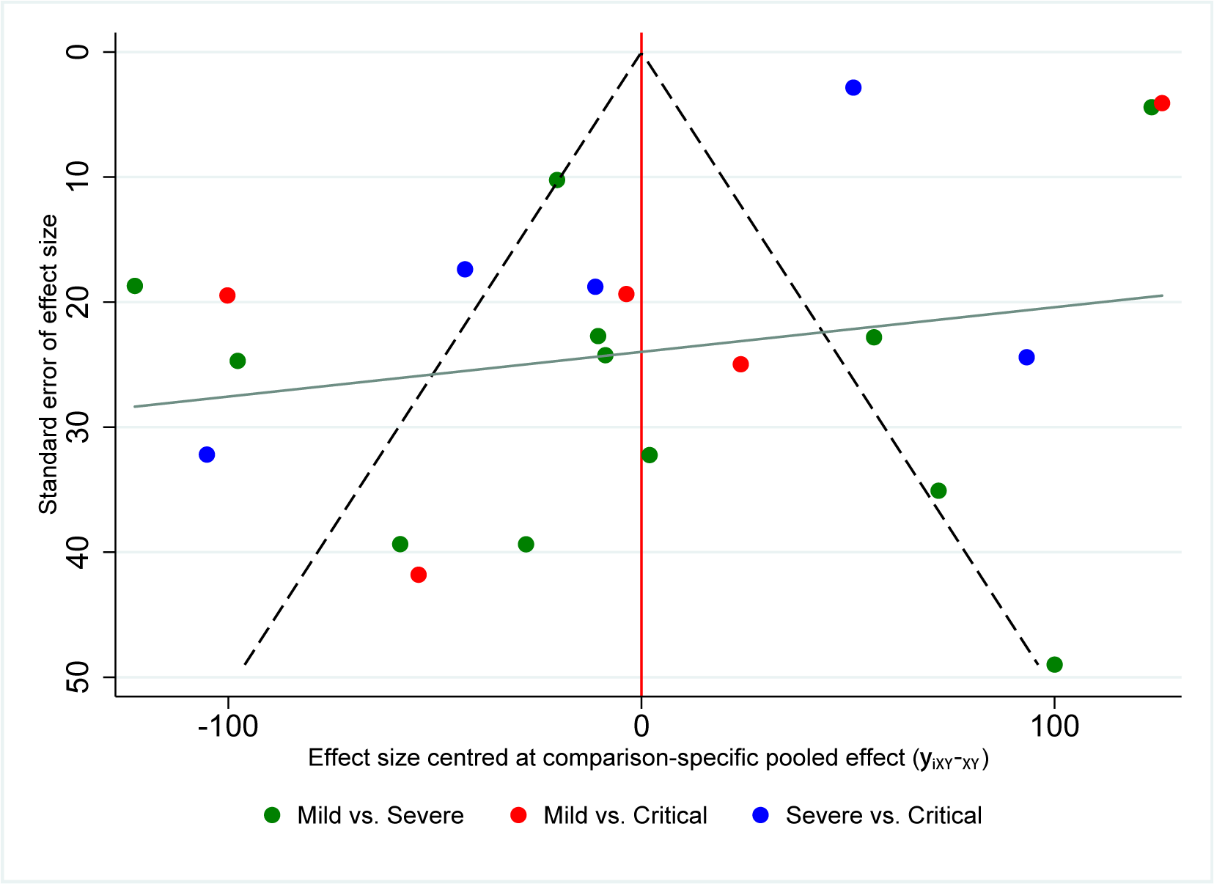
**

**Figure S24** Funnel plot, CD8^+^

**
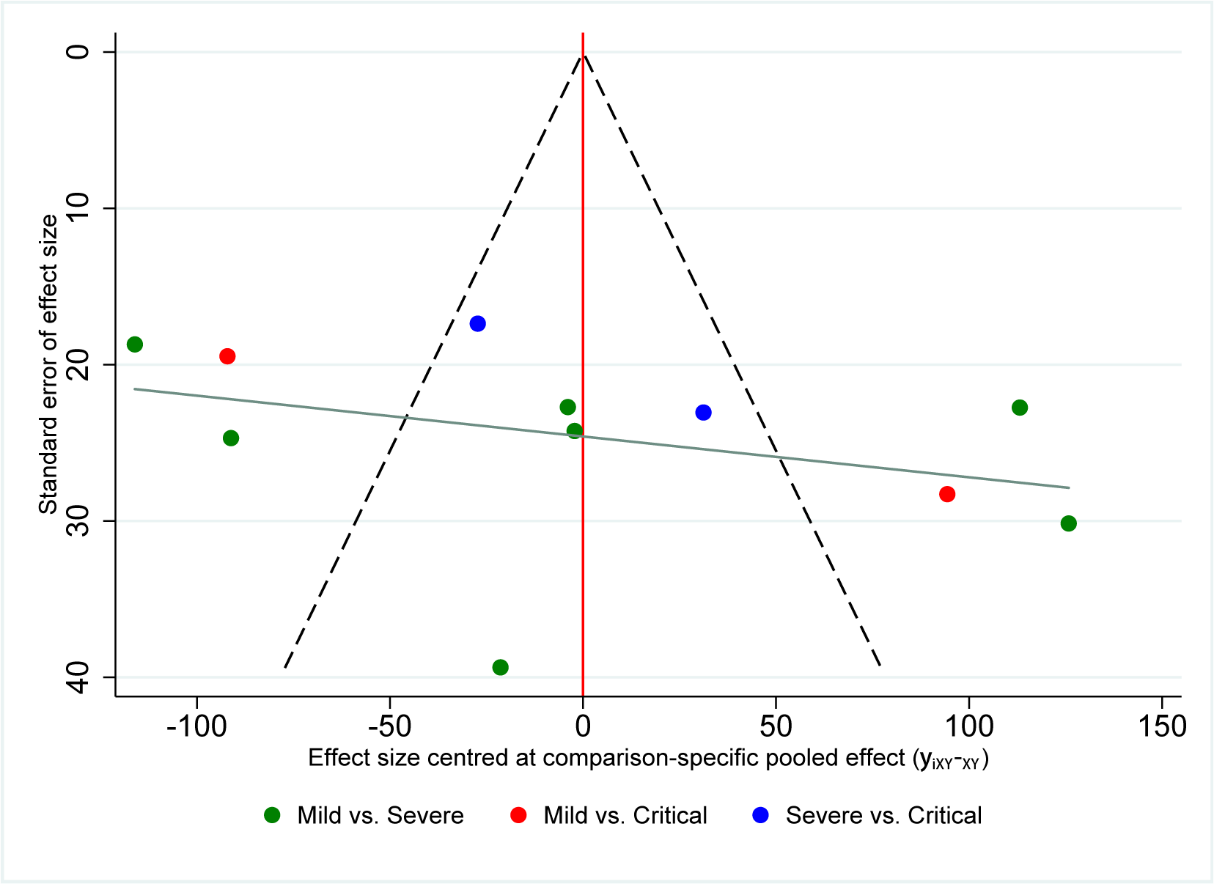
**

**Figure S25** Funnel plot, CD19^+^

**
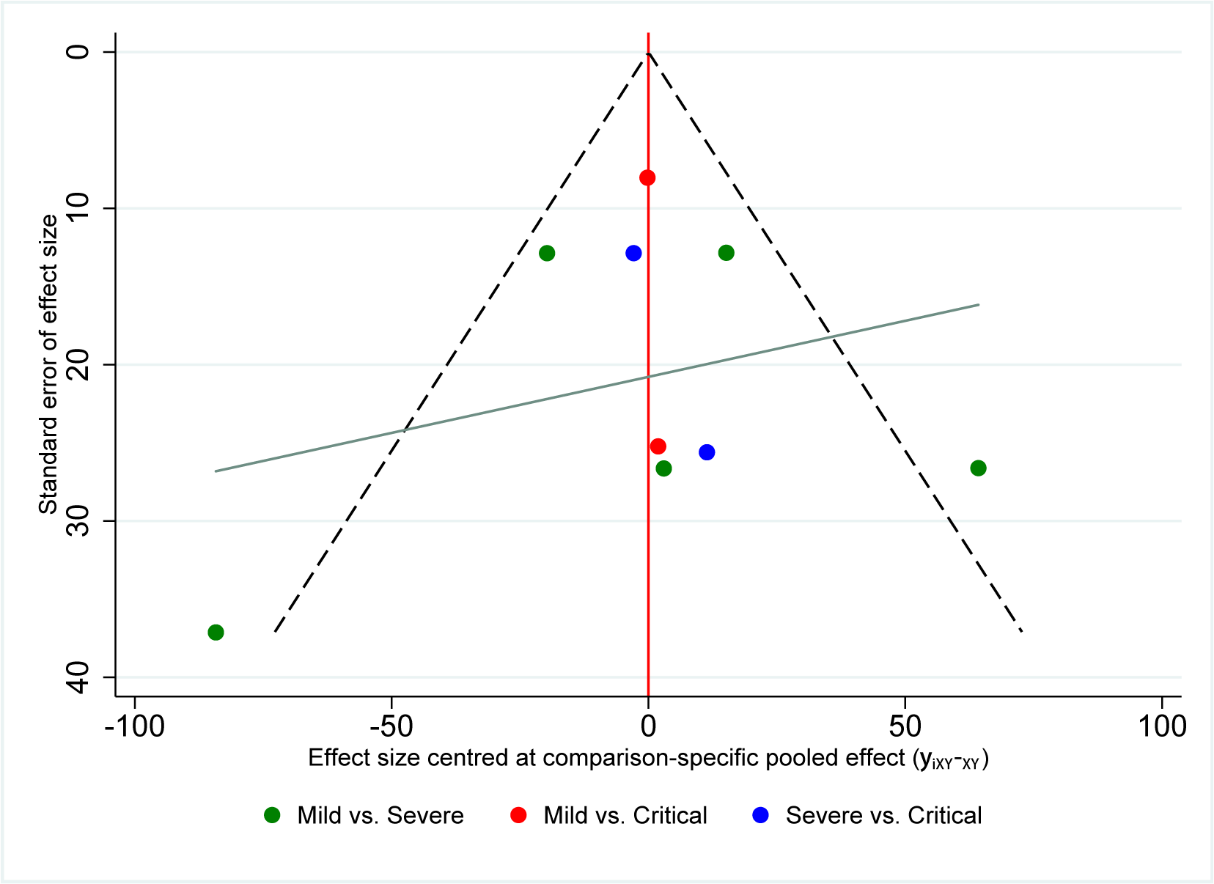
**

**Figure S26** Funnel plot, CD16^+^ CD56^+^

**
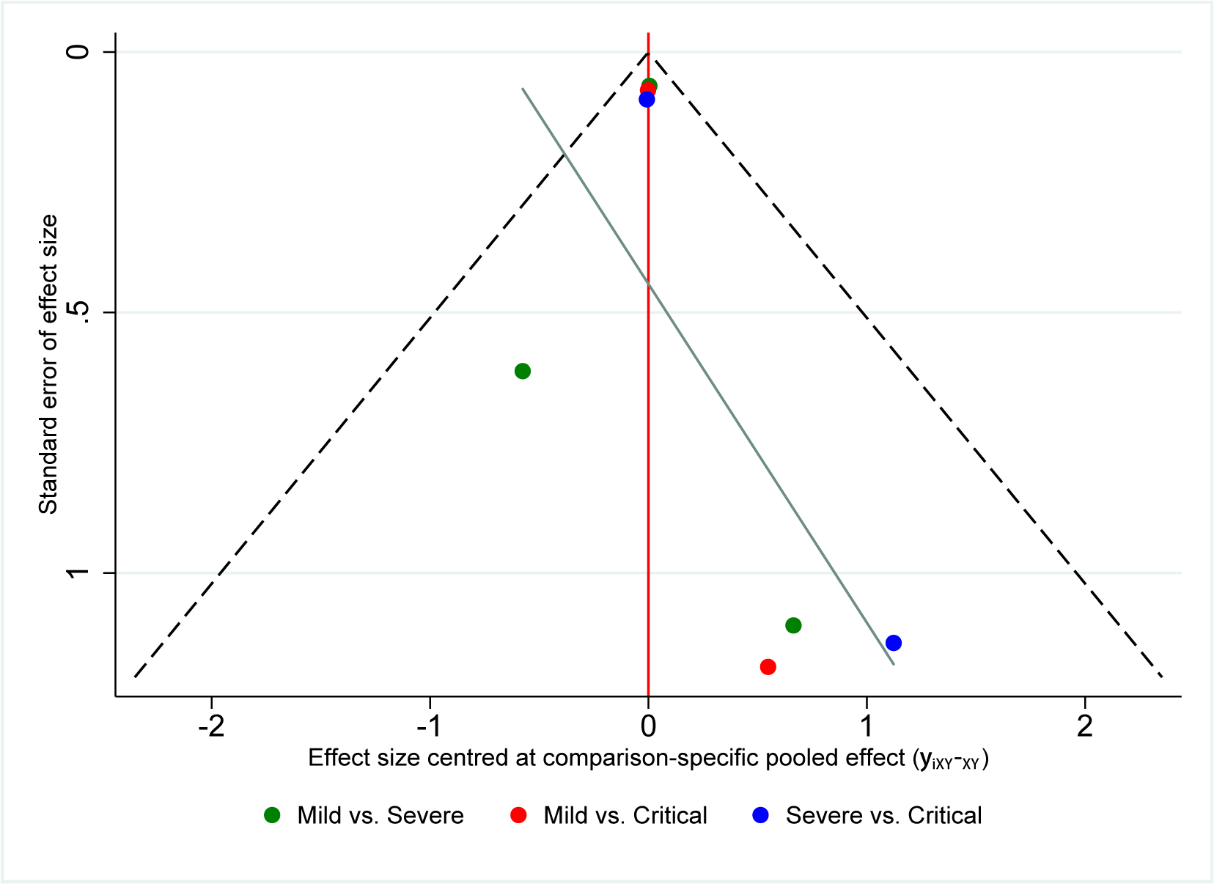
**

**Figure S27** Funnel plot, IL-1β

**
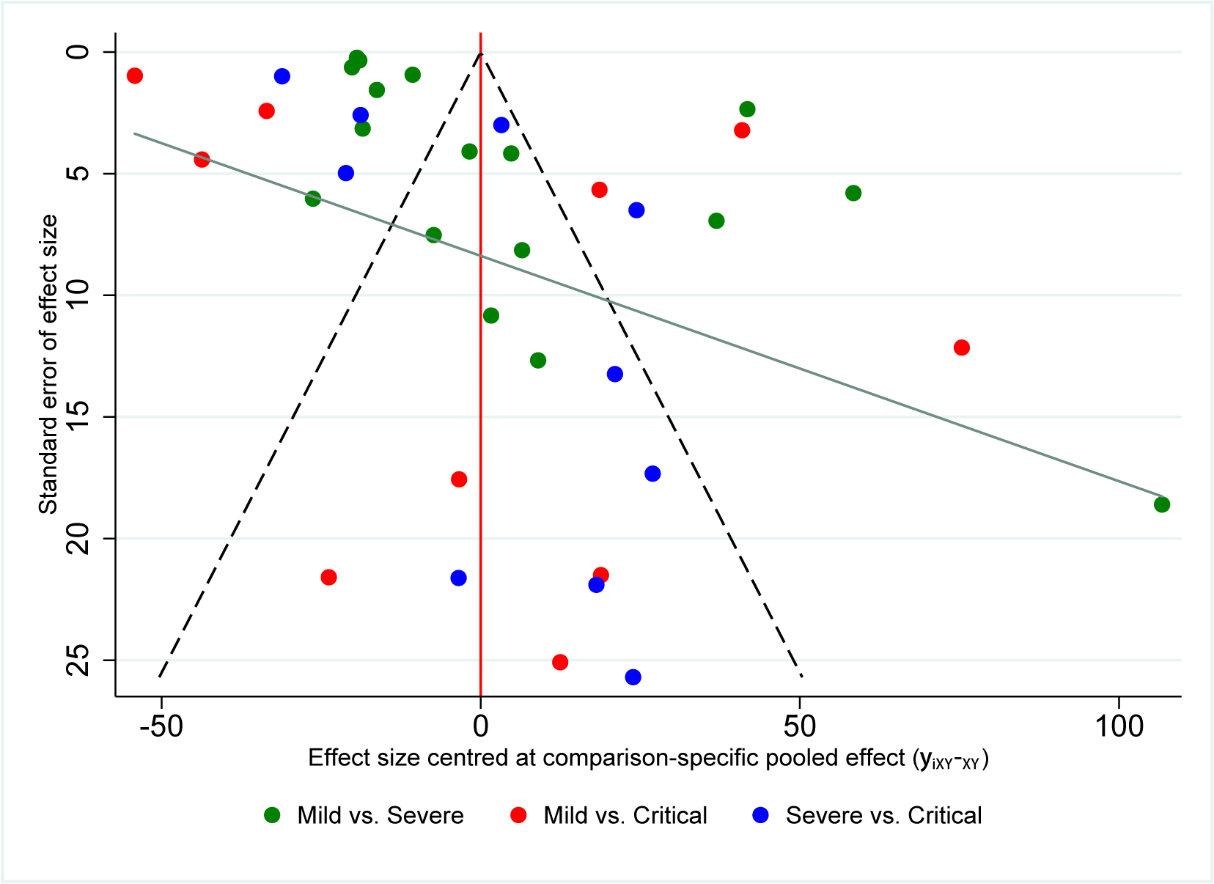
**

**Figure S28** Funnel plot, IL-6

**
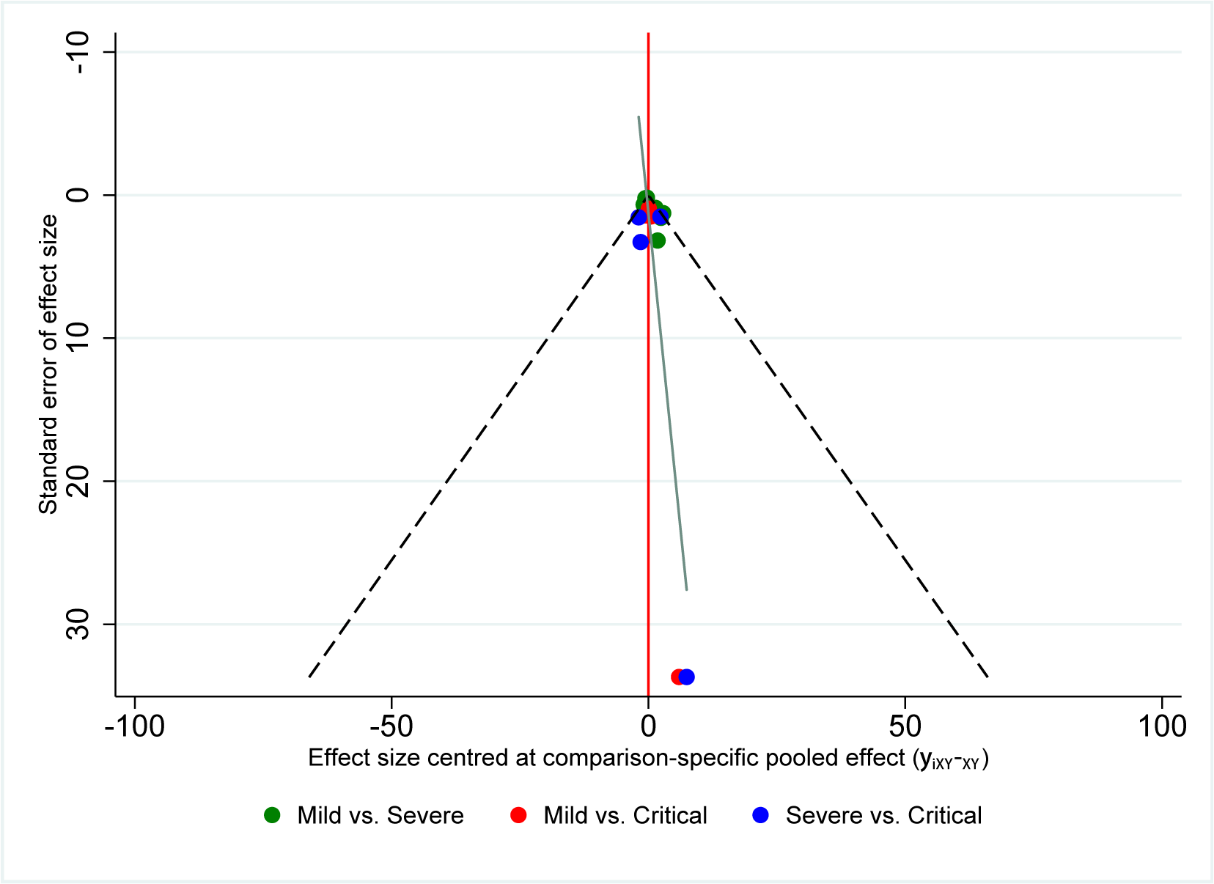
**

**Figure S29** Funnel plot, IL-10

**
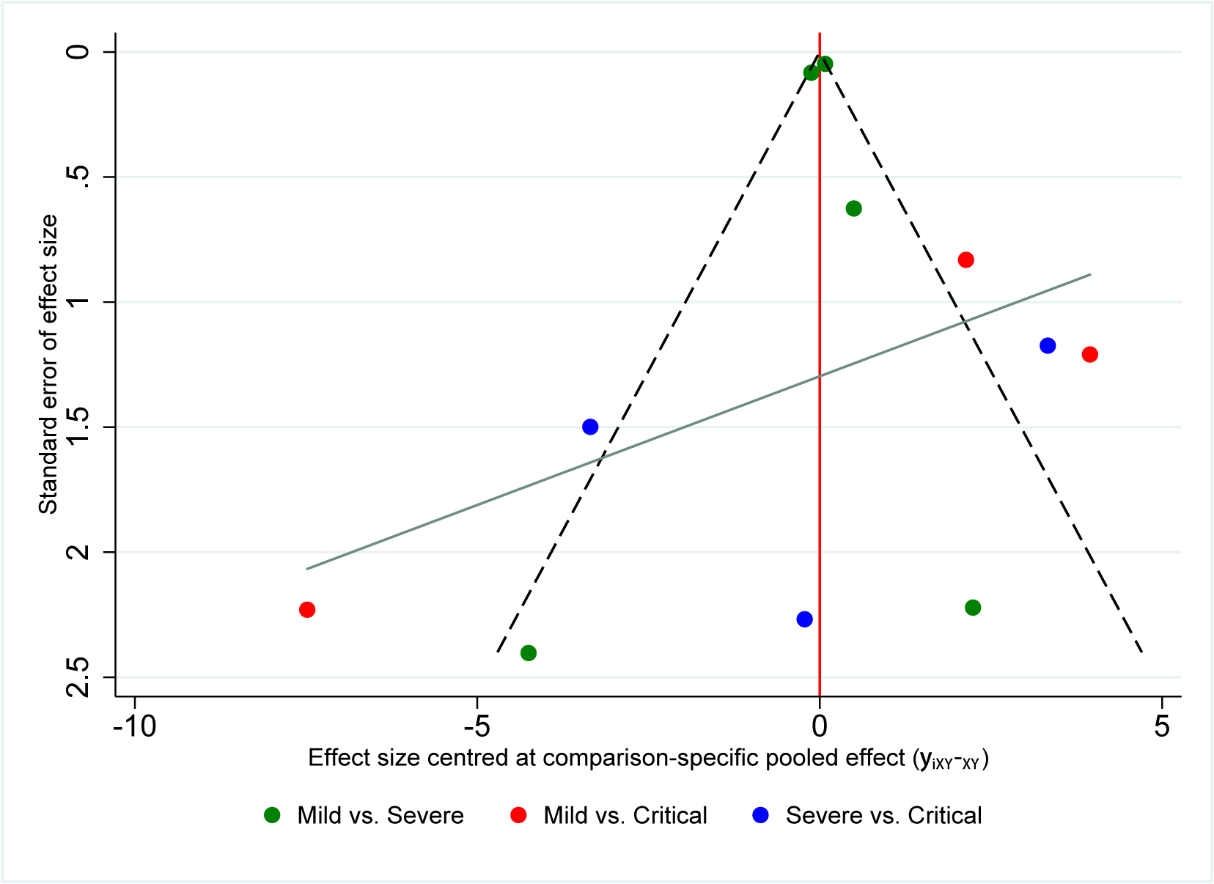
**

**Figure S30** Funnel plot, TNF-α

**Table S4** Subgroup analysis of immune-inflammatory parameters in COVID-19 patients with different clinical types

|  | **Comparisons** | **＜52 years** | | **≥52 years** | |
| --- | --- | --- | --- | --- | --- |
|  |  | **MD** | **95% CI** | **MD** | **95% CI** |
| WBC | Severe VS. Mild | 0.94 | (-2.34, 0.45) | -1.07 | (-2.36, 0.23) |
|  | Critical VS. Mild | **1.24** | **(0.61, 1.87)** | **1.80** | **(0.86, 2.74)** |
|  | Critical VS. Severe | 1.20 | (-0.45, 2.86) | 0.39 | (-0.92, 1.70) |
| LYM | Severe VS. Mild | 0.22 | (-0.04, 0.49) | 0.01 | (-0.15, 0.18) |
|  | Critical VS. Mild | **-0.46** | **(-0.56, -0.35)** | **-0.32** | **(-0.43, -0.21)** |
|  | Critical VS. Severe | -0.04 | (-0.31, 0.22) | **-0.26** | **(-0.42, -0.10)** |
| NEUT | Severe VS. Mild | -1.97 | (-4.05, 0.10) | -0.08 | (-1.65, 1.49) |
|  | Critical VS. Mild | **2.07** | **(1.27, 2.87)** | 0.81 | (-0.43, 2.05) |
|  | Critical VS. Severe | 2.45 | (-0.16, 5.07) | **1.64** | **(0.04, 3.23)** |
| MONO | Severe VS. Mild | -0.10 | (-0.21, 0.00) | 0.04 | (-0.15, 0.23) |
|  | Critical VS. Mild | 0.00 | (-0.06, 0.06) | -0.08 | (-0.23, 0.07) |
|  | Critical VS. Severe | -0.12 | (-0.35, 0.11) | 0.04 | (-0.15, 0.23) |
| PLT | Severe VS. Mild | 0.31 | (-28.99, 29.61) | 3.12 | (-30.84, 37.08) |
|  | Critical VS. Mild | **-13.22** | **(-24.56, -1.88)** | **-32.29** | **(-57.97, -6.60)** |
|  | Critical VS. Severe | -32.05 | (-64.73, 0.63) | 0.81 | (-33.35, 34.97) |
| HB | Severe VS. Mild | **-4.68** | **(-6.39, -2.98)** | **-5.76** | **(-14.07, 2.55)** |
|  | Critical VS. Mild | -4.18 | (-11.46, 3.10) | 3.67 | (-4.12, 11.46) |
|  | Critical VS. Severe | 0.50 | (-6.97, 7.98) | -6.03 | (-16.06, 3.99) |
| CD3^+^ | Severe VS. Mild | **-498.04** | **(-731.80, -264.29)** | **-277.38** | **(-469.21, -85.56)** |
|  | Critical VS. Mild | — |  | **-425.35** | **(-621.97, -228.74)** |
|  | Critical VS. Severe | — |  | -147.97 | (-339.70, 43.76) |
| CD4^+^ | Severe VS. Mild | **-255.00** | **(-350.44, -159.57)** | **-151.22** | **(-238.82, -63.62)** |
|  | Critical VS. Mild | — |  | **-237.71** | **(-333.97, -141.45)** |
|  | Critical VS. Severe | — |  | -86.49 | (-181.59, 8.61) |
| CD8^+^ | Severe VS. Mild | **-171.70** | **(-241.38, -102.02)** | **-89.86** | **(-152.95, -26.76)** |
|  | Critical VS. Mild | — |  | **-139.78** | **(-206.48, -73.07)** |
|  | Critical VS. Severe | — |  | -49.92 | (-115.97,16.13) |
| CD19^+^ | Severe VS. Mild | **-165.71** | **(-308.08, -23.35)** | **-99.51** | **(-198.94, -0.08)** |
|  | Critical VS. Mild | — |  | **-155.97** | **(-269.46, -42.47)** |
|  | Critical VS. Severe | — |  | -55.46 | (-168.99, 56.07) |
| CD16^+^CD56^+^ | Severe VS. Mild | -4.42 | (-86.97, 78.13) | -30.21 | (-75.95, 15.53) |
|  | Critical VS. Mild | — |  | **-60.86** | **(-100.14, -21.59)** |
|  | Critical VS. Severe | — |  | -30.65 | (-71.93, 10.62) |
| IL-1β | Severe VS. Mild | — |  | 0.07 | (-0.05, 0.20) |
|  | Critical VS. Mild | — |  | **0.15** | **(0.01, 0.30)** |
|  | Critical VS. Severe | — |  | 0.08 | (-0.10, 0.26) |
| IL-6 | Severe VS. Mild | **31.20** | **(4.80, 57.60)** | 14.56 | (-3.90, 33.03) |
|  | Critical VS. Mild | 32.90 | (-31.13, 96.93) | **48.53** | **(28.11, 68.95)** |
|  | Critical VS. Severe | 1.70 | (-62.33, 65.74) | **33.97** | **(13.48, 54.46)** |
| IL-10 | Severe VS. Mild | **1.91** | **(0.15, 3.68)** | 1.15 | (-0.42, 2.72) |
|  | Critical VS. Mild | — |  | **3.85** | **(2.08, 5.61)** |
|  | Critical VS. Severe | — |  | **2.70** | **(0.43, 4.96)** |
| TNF-α | Severe VS. Mild | **0.25** | **(0.05 ,0.45)** | 0.10 | (-4.82, 5.02) |
|  | Critical VS. Mild | — |  | -0.37 | (-5.15, 4.42) |
|  | Critical VS. Severe | — |  | -0.47 | (-5.29, 4.36) |

**Note:** WBC: white blood cell; LYM: lymphocyte; NEUT: neutrophil; MONO: Monocytes; PLT: platelet; HB: hemoglobin; CD3^+^: cluster of differentiation 3^+^; CD4^+^: cluster of differentiation 4^+^; CD8^+^: cluster of differentiation 8^+^; CD19^+^: cluster of differentiation 19^+^; CD16^+^56^+^: cluster of differentiation 16^+^56^+^; IL-1β: interlcukin-1β; IL-6: interlcukin-6; IL-10: interlcukin-10; TNF-α: tumor necrosis factor-α. MD: mean difference. 95% CI: 95% confidence interval.

**Table S5** Comparison of direct, indirect, and network meta-analyses results

|  | **Comparisons** | **Direct evidence** | | **Indirect evidence** | |  | **Network meta-analysis** | |
| --- | --- | --- | --- | --- | --- | --- | --- | --- |
|  |  | **MD** | **95% CI** | **MD** | **95% CI** | ***P-*value** | **MD** | **95% CI** |
| WBC | Severe VS. Mild | **1.14** | **(0.72, 1.55)** | -2.63 | (-6.70, 1.45) | 0.072 | **1.10** | **(0.68, 1.53)** |
|  | Critical VS. Mild | **2.00** | **(1.28, 2.72)** | **3.71** | **(2.11, 5.31)** | 0.047 | **2.26** | **(1.57, 2.95)** |
|  | Critical VS. Severe | **1.60** | **(0.83, 2.36)** | -0.36 | (-1.73, 1.00) | 0.012 | **1.16** | **(0.46, 1.86)** |
| LYM | Severe VS. Mild | **-0.38** | **(-0.44, -0.31)** | -0.08 | (-0.77, 0.61) | 0.409 | **-0.37** | **(-0.44, -0.31)** |
|  | Critical VS. Mild | **-0.54** | **(-0.65, -0.42)** | **-0.77** | **(-1.01, -0.54)** | 0.701 | **-0.58** | **(-0.68, -0.48)** |
|  | Critical VS. Severe | **-0.26** | **(-0.38, -0.15)** | 0.02 | (-0.20, 0.24) | 0.026 | **-0.21** | **(-0.31, -0.10)** |
| NEUT | Severe VS. Mild | **1.24** | **(0.69, 1.78)** | -2.49 | (-7.74, 2.77) | 0.167 | **1.2** | **(0.65, 1.75)** |
|  | Critical VS. Mild | **2.45** | **(1.58, 3.33)** | **4.48** | **(2.49, 6.47)** | 0.060 | **2.76** | **(1.92, 3.60)** |
|  | Critical VS. Severe | **2.10** | **(1.1, 2.92)** | -0.34 | (-2.11, 1.44) | 0.019 | **1.56** | **(0.70, 2.42)** |
| MONO | Severe VS. Mild | -0.03 | (-0.08, 0.01) | 0.03 | (-0.25, 0.32) | 0.644 | -0.03 | (-0.08, 0.01) |
|  | Critical VS. Mild | -0.05 | (-0.12, 0.02) | 0.00 | (-0.17, 0.16) | 0.618 | -0.04 | (-0.10, 0.02) |
|  | Critical VS. Severe | -0.01 | (-0.08, 0.07) | -0.03 | (-0.17, 0.10) | 0.720 | -0.01 | (-0.08, 0.06) |
| PLT | Severe VS. Mild | **-19.21** | **(-28.24, -10.18)** | 15.57 | (-72.84, 103.98) | 0.443 | **-18.87** | **(-27.86, -9.88)** |
|  | Critical VS. Mild | **-31.5** | **(-47.91, -15.09)** | -22.02 | (-57.97, 13.92) | 0.629 | **-30.13** | **(-45.24, -15.03)** |
|  | Critical VS. Severe | **-10.40** | **(-28.36, 7.56)** | -13.54 | (-44.56, 17.48) | 0.862 | **-11.26** | **(-26.74, 4.21)** |
| HB | Severe VS. Mild | **-3.86** | **(-5.52, -2.2)** | -1.35 | (-17.59, 14.89) | 0.764 | **-3.81** | **(-5.43, -2.19)** |
|  | Critical VS. Mild | -3.38 | (-8.04, 1.28) | **-8.13** | **(-15.46, -0.79)** | 0.147 | -3.99 | (-8.75, 0.76) |
|  | Critical VS. Severe | -2.01 | (-7.6, 3.58) | **1.73** | **(-3.93, 7.39)** | 0.227 | -0.18 | (-5.04, 4.67) |
| CD3^+^ | Severe VS. Mild | — |  | — |  |  | **-389.02** | **(-549.59, -228.45)** |
|  | Critical VS. Mild | **-423.02** | **(-637, -209.04)** | **-870.47** | **(-1417.79, -323.14)** | 0.135 | **-479.87** | **(-696.64, -263.10)** |
|  | Critical VS. Severe | -146.87 | (-356.7, 62.96) | 300.58 | (-250.84, 852.00) | 0.135 | **-90.85** | **(-305.37, 123.67)** |
| CD4^+^ | Severe VS. Mild | — |  | — |  |  | **-198.74** | **(-262.12, -135.35)** |
|  | Critical VS. Mild | **-227.15** | **(-324.15, -130.15)** | **-446.23** | **(-671.17, -221.29)** | 0.081 | **-262.18** | **(-356.76, -167.60)** |
|  | Critical VS. Severe | **-96.37** | **(-192.02, -0.72)** | 122.72 | (-104.00, 349.43) | 0.081 | -63.44 | (-157.51, 30.62) |
| CD8^+^ | Severe VS. Mild | — |  | — |  |  | **-127.49** | **(-173.75, -81.24)** |
|  | Critical VS. Mild | **-133.82** | **(-198.22, -69.42)** | -306.03 | (-464.24, -147.82) | **0.049** | **-159.06** | **(-223.91, -94.22)** |
|  | Critical VS. Severe | -55.28 | (-118.94, 8.38) | 116.93 | (-42.23, 276.09) | 0.049 | -31.57 | (-96.09, 32.95) |
| CD19^+^ | Severe VS. Mild | — |  | — |  |  | **-134.32** | **(-203.92, -64.71)** |
|  | Critical VS. Mild | **-142.65** | **(-270.78, -14.51)** | **-311.39** | **(-586.68, -36.10)** | 0.279 | **-173.38** | **(-289.84, -56.91)** |
|  | Critical VS. Severe | -68.694 | (-195.53, 58.14) | 100.05 | (-177.04, 377.13) | 0.279 | -39.06 | (-155.12, 76.99) |
| CD16^+^CD56^+^ | Severe VS. Mild | — |  | — |  |  | -23.27 | (-55.79, 9.25) |
|  | Critical VS. Mild | -52.92 | (-108.94, 3.11) | -89.52 | (-231.48, 52.44) | 0.639 | **-57.68** | **(-101.37, -14.00)** |
|  | Critical VS. Severe | -39.42 | (-96.73, 17.89) | -2.82 | (-143.35, 137.72) | 0.639 | -34.41 | (-78.91, 10.08,) |
| IL-1β | Severe VS. Mild | — |  |  |  |  | 0.07 | (-0.05, 0.20) |
|  | Critical VS. Mild | **0.15** | **(0.01, 0.3)** | 1.49 | (-2.83, 5.81) | 0.545 | **0.15** | **(0.01, 0.30)** |
|  | Critical VS. Severe | **0.81** | **(0.63, 0.98)** | -1.26 | (-5.57, 3.06) | 0.545 | 0.08 | (-0.10, 0.26) |
| IL-6 | Severe VS. Mild | — |  | — |  |  | -13.75 | (-46.31, 18.82) |
|  | Critical VS. Mild | **54.27** | **(32.47, 76.06)** | **81.76** | **(19.37, 144.15)** | 0.408 | **34.05** | **(8.70, 59.39)** |
|  | Critical VS. Severe | **33.97** | **(12.11, 55.83)** | 6.47 | (-55.75, 68.7) | 0.408 | 20.22 | (-13.16, 53.60) |
| IL-10 | Severe VS. Mild | — |  |  |  |  | -0.68 | (-2.71, 1.34) |
|  | Critical VS. Mild | **3.84** | **(2.02, 5.65)** | **2.15** | **(0.99, 9.41)** | 0.510 | **1.88** | **(0.26, 3.49)** |
|  | Critical VS. Severe | **2.64** | **(0.49, 4.80)** | 2.02 | (-2.68, 5.24) | 0.510 | 1.96 | (-0.50, 4.42) |
| TNF-α | Severe VS. Mild | — |  | — |  |  | -0.03 | (-6.24, 6.18) |
|  | Critical VS. Mild | -0.23 | (-4.33, 3.87) | -0.17 | (-11.83, 11.49) | 0.992 | 0.24 | (-4.30, 4.78) |
|  | Critical VS. Severe | -0.44 | (-4.58, 3.7) | -0.50 | (-12.12, 11.12) | 0.992 | -0.47 | (-6.59, 5.65) |

Note: WBC: white blood cell; LYM: lymphocyte; NEUT: neutrophil; MONO: Monocytes; PLT: platelet; HB: hemoglobin; CD3^+^: cluster of differentiation 3^+^; CD4^+^: cluster of differentiation 4^+^; CD8^+^: cluster of differentiation 8^+^; CD19^+^: cluster of differentiation 19^+^; CD16^+^56^+^: cluster of differentiation 16^+^56^+^; IL-1: interlcukin-1; IL-6: interlcukin-6; IL-10: interlcukin-10; TNF-α: tumor necrosis factor-α. MD: mean difference. 95% CI: 95% confidence interval.

**References**

1 Zhu Z, Cai T, Fan L, Lou K, Hua X, Huang Z, et al. Clinical value of immune-inflammatory parameters to assess the severity of coronavirus disease 2019. Int J Infect Dis 2020;95:332-9.

2 Zhou Y, Han T, Chen J, Hou C, Hua L, He S, et al. Clinical and Autoimmune Characteristics of Severe and Critical Cases of COVID-19. Clin Transl Sci 2020.

3 Diao B, Wang C, Tan Y, Chen X, Liu Y, Ning L, et al. Reduction and Functional Exhaustion of T Cells in Patients with Coronavirus Disease 2019 (COVID-19). medRxiv 2020:2020.02.18.20024364.

4 Chen G, Wu D, Guo W, Cao Y, Huang D, Wang H, et al. Clinical and immunological features of severe and moderate coronavirus disease 2019. J Clin Invest 2020;130:2620-9.

5 Chen X, Zhao B, Qu Y, Chen Y, Xiong J, Feng Y, et al. Detectable serum SARS-CoV-2 viral load (RNAaemia) is closely correlated with drastically elevated interleukin 6 (IL-6) level in critically ill COVID-19 patients. Clin Infect Dis 2020.

6 Gao Y, Li T, Han M, Li X, Wu D, Xu Y, et al. Diagnostic utility of clinical laboratory data determinations for patients with the severe COVID-19. J Med Virol 2020.

7 He R, Lu Z, Zhang L, Fan T, Xiong R, Shen X, et al. The clinical course and its correlated immune status in COVID-19 pneumonia. J Clin Virol 2020;127:104361.

8 Huang C, Wang Y, Li X, Ren L, Zhao J, Hu Y, et al. Clinical features of patients infected with 2019 novel coronavirus in Wuhan, China. Lancet 2020;395:497-506.

9 Liu J, Li S, Liu J, Liang B, Wang X, Wang H, et al. Longitudinal characteristics of lymphocyte responses and cytokine profiles in the peripheral blood of SARS-CoV-2 infected patients. EBioMedicine 2020;55:102763.

10 Liu Y, Liao W, Wan L, Xiang T, Zhang W. Correlation Between Relative Nasopharyngeal Virus RNA Load and Lymphocyte Count Disease Severity in Patients with COVID-19. Viral Immunol 2020.

11 Qu R, Ling Y, Zhang YH, Wei LY, Chen X, Li XM, et al. Platelet-to-lymphocyte ratio is associated with prognosis in patients with coronavirus disease-19. Journal of Medical Virology 2020:1-9.

12 Sun S, Cai X, Wang H, He G, Lin Y, Lu B, et al. Abnormalities of peripheral blood system in patients with COVID-19 in Wenzhou, China. Clin Chim Acta 2020;507:174-80.

13 Wan S, Yi Q, Fan S, Lv J. Relationships among lymphocyte subsets, cytokines, and the pulmonary inflammation index in coronavirus (COVID-19) infected patients. British Journal of Haematology 2020;189:428-37.

14 Wang F, Hou H, Luo Y, Tang G, Wu S, Huang M, et al. The laboratory tests and host immunity of COVID-19 patients with different severity of illness. JCI Insight 2020.

15 Xia XY, Wu J, Liu HL, Xia H, Jia B, Huang WX. Epidemiological and initial clinical characteristics of patients with family aggregation of COVID-19. J Clin Virol 2020;127:104360.

16 Zheng F, Tang W, Li H, Huang YX, Xie YL, Zhou ZG. Clinical characteristics of 161 cases of corona virus disease 2019 (COVID-19) in Changsha. Eur Rev Med Pharmacol Sci 2020;24:3404-10.

17 Zhang JJ, Dong X, Cao YY, Yuan YD, Yang YB, Yan YQ, et al. Clinical characteristics of 140 patients infected with SARS-CoV-2 in Wuhan, China. Allergy 2020.

18 Zhang G, Hu C, Luo L, Fang F, Chen Y, Li J, et al. Clinical features and short-term outcomes of 221 patients with COVID-19 in Wuhan, China. J Clin Virol 2020;127:104364.

19 Yao Q, Wang P, Wang X, Qie G, Meng M, Tong X, et al. Retrospective study of risk factors for severe SARS-Cov-2 infections in hospitalized adult patients. Pol Arch Intern Med 2020.

20 Xu B, Fan CY, Wang AL, Zou YL, Yu YH, He C, et al. Suppressed T cell-mediated immunity in patients with COVID-19: A clinical retrospective study in Wuhan, China. J Infect 2020.

21 Xie H, Zhao J, Lian N, Lin S, Xie Q, Zhuo H. Clinical characteristics of non-ICU hospitalized patients with coronavirus disease 2019 and liver injury: A retrospective study. Liver Int 2020.

22 Chang Z, Yang W, Wang Q, Liao G. Clinical significance of serum hs-CRP, IL-6, and PCT in diagnosis and prognosis of patients with COVID-19. Drugs & Clinic 2020;35.

23 Chen L, Liu H, Liu W, Liu J, Liu K, Shang J, et al. Analysis of clinical features of 29 patients with 2019 novel coronaviruia pneumonia. Chinese Journal of Tuberculosis and Respiratory Diseases 2020:203-8.

24 Chen M, An W, Xia F, Yang P, Liao Y, Fang S, et al. Retrospective Analysis of COVID-19 Patients with Different Clinical Subtypes. Herald of Medicine 2020;39:459-64.

25 Chen S, Wu J, Li Z, Xu D, Zhu Z, Wang C, et al. Clinical features of 109 cases of novel coronavirus pneumonia. Chinese Journal of Infectious Diseases 2020;38.

26 Chen W, Xu L, Zhang Q, Li L, Lu C. Clinical characteristics of 91 novel coronavirus pneumonia patients in Jingmen First People's Hospital. Journal of Inner Mongolia Medical University 2020:1-15.

27 Chen X, Tong J, Xiang J, Hu J. Retrospective study on the epidemiological characteristics of 139 patients with novel coronavirus pneumonia on the effects of Severity. Chongqing Medicine 2020:1-9.

28 Chen X, Qu J, Huang Y, Tan M, Chen J, Lin L, et al. Diagnostic roles of several parameters in corona virus disease 2019. Laboratory Medicine 2020:1-8.

29 Cheng K, Wei M, Shen H, Wu C, Chen D, Xiong W, et al. Clinical characteristics of 463 patients with common and severe type coronavirus disease. Shanghai Medical Journal:1-15.

30 Fang X, Mei Q, Yang T, Zhang L, Yang Y, Wang Y, et al. Clinical characteristics and treatment strategies of 79 patients with COVID-19. Chinese Pharmacological Bulletin 2020;36:453-9.

31 Lei Y, Lu J, Gu J, Zhou D, Li Y, Lu Q. Clinical features of 51 patients with corona virus disease 2019 in Wuhan City. Journal of Shandong University(Health Sciences):1-6.

32 Li D, Liu H, Wang Y, Guo H, Wang Y, Wang K, et al. Clinical features of 30 cases with novel coronavirus pneumonia. Chinese Journal of Infectious Diseases 2020;38.

33 Li D, Long Y, Huang P, Guo W, Wu S, Zhou Q, et al. Clinical characteristics of 80 patients with COVID-19 in Zhuzhou City. Chinese Journal of Infection Control 2020;19:227-33.

34 Li D, Wang M, He B, Xu Y, Zhou X, Li W, et al. Laboratory test analysis of 62 COVID-19 patients. Medical Journal of Wuhan University:1-5.

35 Liu M, He P, Liu H, Wang X, Li F, Chen S, et al. Clinical characteristics of 30 medical workers infected with new coronavirus pneumonia. Chinese Journal of Tuberculosis and Respiratory Diseases 2020:209-14.

36 Liu S, Cheng F, Yang X, He J, Li H, Zhang W, et al. A study of laboratory confirmed cases between laboratory indexes and clinical classification of 342 cases with Corona Virus Disease 2019 in Ezhou. Laboratory Medicine:1-12.

37 Liu Y, Yang Y, Zhang C, Huang F, Wang F, Yuan J, et al. Clinical and biochemical indexes from 2019-nCoV infected patients linked to viral loads and lung injury. Science China Life Sciences 2020;50:258-69.

38 Peng Y, Meng K, Guan H, Leng L, Zhu R, Wang B, et al. Clinical characteristics and outcomes of 112 cardiovascular disease patients infected by 2019-nCoV. Chinese Journal of Cardiology 2020;48.

39 Song X, Chen T, Sun J, Chen M, Liu W, Wu M, et al. Discussion on the clinical value of PCT combined with IL-6 in novel coronavirus pneumonia. International Journal of Laboratory Medicine:1-8.

40 Tang J, Xuan C, Lin T, Zhang E, Zhong M, Deng D. Clinical significance of detecting c-reactive protein, interleukin-6 and procalcitonin in COVID-19. The Journal of Practical Medicine 2020;36:839-41.

41 Wan Q, Shi A, He T, Tang L. Analvsis of clinical features of 153 patients with novel coronavirus pneumonia in Chongging. Chinese Journal of Clinical Infectious Diseases 2020;13.

42 Xiang T, Liu J, Xu F, Cheng N, Liu Y, Qian K, et al. Analysis of clinical characteristics of 49 patients with coronavirus disease 2019 in Jiangxi. Chinese Journal of Respiratory and Critical Care Medicine 2020;19:154-60.

43 Xiao K, Shui L, Pang X, Mou H, Wang J, Lang C, et al. The clinical features of the 143 patients with COVID-19 in North-East of Chongqing. Journal of Third Military Medical University 2020;42:549-54.

44 Xiong J, Jiang W, Zhou J, Hu X, Liu C. Clinical characteristics, treatment, and prognosis in 89 cases of OVID-2019. Medical Journal of Wuhan University 2020:1-5.

45 Xu J, Han M, Zhao F, Zhang T, Ma L. Clinical manifestations and sero-immunological characteristics of 155patientswithCOVID-19. Chinese Journal of Nosocomiology:1-5.

46 Yuan J, Sun Y, Zuo Y, Chen T, Cao Q, Yuan G, et al. A Retrospective Analysis of the Clinical Characteristics of 223 NCP Patients in Chongqing. Journal of Southwest University(Natural Science Edition) 2020;42:17-24.

47 Zhan T, Zheng H, Mai Y, Li W, Zhuo Y. The correlation between serological dynamic evolution and the severity of coronavirus disease 2019. Guangdong Medical Journal:1-6.

48 Zuo F, Li C, Dong Z, Chang X, Jia M, Wei C, et al. Analysis of the correlation between clinical characteristics and disease severity in patients with novel coronavirus pneumonia. Tianjin Medical Journal:1-6.

49 Zou Q, Lin F, Wang Y, Xing H, Tan J, Zhang Y, et al. Changes and clinical significance of serum IL-2R, IL-6 and TNF-a in elderly patients with COVID-19. Journal of Changchun University of Chinese Medicine 2020.

50 Zhong S, Lin F, Shi L. The clinical characteristics and outcome of 62 patients with COVID-19. Medical Journal of Chinese People's Liberation Army:1-9.

51 Zhang W, Hou W, Li T, Li A, Pan W, Jin R, et al. Clinical characteristics of 74 hospitalized patients with COVID-19. Journal of Capital Medical University 2020;41:161-7.

52 Yan B, Ji P, Liang X, Liu G, Zhang J. Clinical characteristics of 55 hospitalized patients with COVID-19 in Wuhan, China. Journal of Guangxi Medical University 2020;37.

53 Guo N, Huang S, Hu X, Ge L, Liang L, Luo Y. Preliminary study on myocardial injury markers in patients with novel coronavirus pneumonia. Journal of Cardiovascular & Pulmonary Diseases 2020;39:366-9.

54 Huang Y, Xie J, Zang J, Xie Y, Zheng F, Tang W, et al. Analysis of 121 novel coronavirus pneumonia cases. The Journal of Practical Medicine 2020.

55 Li G, Li L, He M, Lin H, Ke P, Zhong Z, et al. Value of various inflammatory markers combined with lymphocyte subsets on clinical diagnosis of different clinical types of COVID-19. Journal of Chongqing Medical University 2020.

56 Li R, Nie H, Wu X. Clinical features of coronavrius disease 2019 with leukocytosis. Medical Journal of Wuhan University 2020.

57 Li R, Tao J, Yao X, Yang F, Peng P, Tang J, et al. Multi-Center Clinical Research of Risk Factors Associated with Severe and Critical atientth Coronavirus Disease 2019. China Pharmaceuticals 2020;29:15-8.

58 Shi Y, Qu J, Chen X, Tan M, Li F, Liu Y. Expressions of multiple inflammation markers in the patients with COVID-19 and their clinical values. Chinese Journal of Laboratory Medicine 2020;43:346-51.

59 Wang D, Li W, Jin F, Wang S, Sun J, Zhou C, et al. Clinical values of laboratory inflammation indicators for diagnosis and treatment of COVID-19 patients. Chinese Journal of Experimental and Clinical Virology 2020;34.

60 Xie Z, Wang L, Li L, Li Y, Guan Y. The predictors for severe coronavirus disease 2019 pneumonia. Guangdong Medical Journal 2020.

61 Ji M, Yuan L, Shen W, Lv J, Li Y, Li M, et al. Characteristics of disease progress in patients with coronavirus disease 2019 in Wuhan, China. 2020;148:e94.

62 Jiang Y, He S, Zhang C, Wang X, Chen X, Jin Y, et al. Clinical characteristics of 60 discharged cases of 2019 novel coronavirus-infected pneumonia in Taizhou, China. Br J Haematol 2020;8:547.

63 Lei S, Jiang F, Su W, Chen C, Chen J, Mei W, et al. Clinical characteristics and outcomes of patients undergoing surgeries during the incubation period of COVID-19 infection. EClinicalMedicine 2020;21.

64 Lv Z, Cheng S, Le J, Huang J, Feng L, Zhang B, et al. Clinical characteristics and co-infections of 354 hospitalized patients with COVID-19 in Wuhan, China: a retrospective cohort study. Microbes and infection 2020.

65 Wang R, Pan M, Zhang X, Han M, Fan X, Zhao F, et al. Epidemiological and clinical features of 125 Hospitalized Patients with COVID-19 in Fuyang, Anhui, China. International Journal of Infectious Diseases 2020;95:421-8.

66 Yang P, Wang P, Song Y, Zhang A, Yuan G, Cui Y. A retrospective study on the epidemiological characteristics and establishment of early warning system of severe COVID-19 patients. Journal of medical virology 2020.

67 Yang Q, Xie L, Zhang W, Zhao L, Wu H, Jiang J, et al. Analysis of the clinical characteristics, drug treatments and prognoses of 136 patients with coronavirus disease 2019. J Clin Pharm Ther 2020.

68 Chen R, Sang L, Jiang M, Yang Z, Jia N, Fu W, et al. Longitudinal hematologic and immunologic variations associated with the progression of COVID-19 patients in China. The Journal of allergy and clinical immunology 2020.

69 Ding X, Yu Y, Lu B, Huo J, Chen M, Kang Y, et al. Dynamic profile and clinical implications of hematological parameters in hospitalized patients with coronavirus disease 2019. Clin Chem Lab Med 2020.

70 Fu J, Kong J, Wang W, Wu M, Yao L, Wang Z, et al. The clinical implication of dynamic neutrophil to lymphocyte ratio and D-dimer in COVID-19: A retrospective study in Suzhou China. Thromb Res 2020;192:3-8.

71 Hong KS, Lee KH, Chung JH, Shin K-C, Choi EY, Jin HJ, et al. Clinical Features and Outcomes of 98 Patients Hospitalized with SARS-CoV-2 Infection in Daegu, South Korea: A Brief Descriptive Study. Yonsei Medical Journal 2020;61:431-7.
